# Direct terrestrial–marine correlation demonstrates surprisingly late onset of the last interglacial in central Europe

**DOI:** 10.1016/j.yqres.2010.11.003

**Published:** 2011-01

**Authors:** Mark J. Sier, Wil Roebroeks, Corrie C. Bakels, Mark J. Dekkers, Enrico Brühl, Dimitri De Loecker, Sabine Gaudzinski-Windheuser, Norbert Hesse, Adam Jagich, Lutz Kindler, Wim J. Kuijper, Thomas Laurat, Herman J. Mücher, Kirsty E.H. Penkman, Daniel Richter, Douwe J.J. van Hinsbergen

**Affiliations:** a Faculty of Archaeology, Leiden University, P.O. Box 9515, 2300 RA Leiden, The Netherlands; b Paleomagnetic Laboratory ‘Fort Hoofddijk’, Department of Earth Sciences, Faculty of Geosciences, Utrecht University, Budapestlaan 17, 3584 CD Utrecht, The Netherlands; c National Center for Human Evolution (CENIEH), Paseo Sierra de Atapuerca s/n, 09002 Burgos, Spain; d Landesamt für Denkmalpflege und Archäologie, Richard-Wagner-Str. 9, 06114 Halle, Germany; e Römisch-Germanisches Zentralmuseum, Forschungsbereich Altsteinzeit, Schloss Monrepos, 56567 Neuwied, Germany; f Johannes Gutenberg-Universität Mainz, Institut für Vor- und Frühgeschichte, Schönborner Hof, Schillerstrasse 11, 55116 Mainz, Germany; g Prinses Beatrixsingel 21, 6301 VK Valkenburg, The Netherlands; h “BioArCh” Department of Chemistry, University of York, Heslington, York, YO10 5DD, UK; i Max Planck Institute for Evolutionary Anthropology, Department of Human Evolution, Deutscher Platz 6, 04103 Leipzig, Germany; j Physics of Geological Processes, University of Oslo, Sem Sælands vei 24, 0391 Oslo, Norway

**Keywords:** Blake Event, Eemian, Last interglacial, MIS 5e, Palaeomagnetism

## Abstract

An interdisciplinary study of a small sedimentary basin at Neumark Nord 2 (NN2), Germany, has yielded a high-resolution record of the palaeomagnetic Blake Event, which we are able to place at the early part of the last interglacial pollen sequence documented from the same section. We use this data to calculate the duration of this stratigraphically important event at 3400 ± 350 yr. More importantly, the Neumark Nord 2 data enables precise terrestrial–marine correlation for the Eemian stage in central Europe. This shows a remarkably large time lag of ca. 5000 yr between the MIS 5e ‘peak’ in the marine record and the start of the last interglacial in this region.

## Introduction

**Figure 1:**
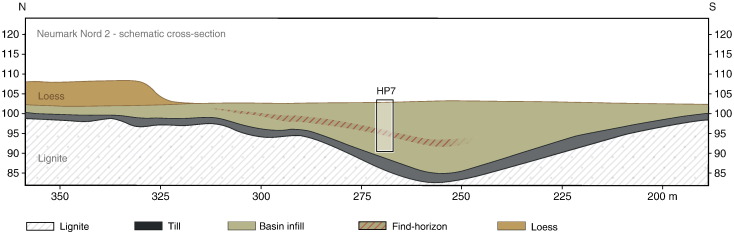
Schematic north–south cross section of the Neumark-Nord 2 basin and its infill including the stratigraphic position of the archaeological find horizon. The box inset refers to the HP7 section (Fig. 2), described in detail in the Supplemental material. Vertical axis: height in meters above sea level. Horizontal axis: position of the NN2 basin in excavation grid, in meters.

Large scale excavations of the Middle Palaeolithic site Neumark Nord 2 (NN2), Germany, carried out between 2004 and 2008, yielded a rich archaeological assemblage, containing ca. 20,000 Middle Palaeolithic flint artifacts and approximately 120,000 faunal remains, dominated by warm-temperate species. The fauna includes straight tusked elephants, rhinoceroses, bovids, equids, deer, bear, small carnivores and the pond tortoise *Emys orbicularis*. Excavations took place in an open cast lignite quarry, south of Halle (Germany), where the archaeology was contained within the infill of a small and shallow sedimentary basin, resulting from diapirism-related movements in the underlying Tertiary lignite deposits (Eissmann, 2002, Mania and Mania, 2008) (Fig. 1). In order to develop a fine-grained palaeoenvironmental and chronological framework for the unique archaeological record from the site, the basin infill was studied using a wide range of techniques. These interdisciplinary studies yielded climatic and chronological proxy records which are of great relevance to the study of the last interglacial and more importantly, enable precise terrestrial–marine correlation for the Eemian stage in central and northwestern Europe.

## The Neumark Nord 2 basin

**Figure 2:**
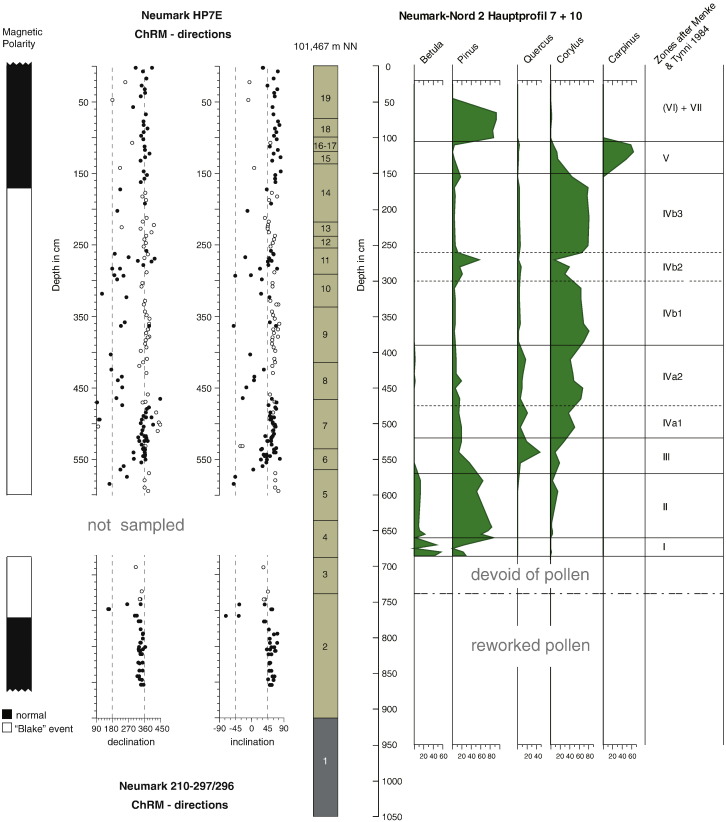
Combined overview of the palaeomagnetic, stratigraphic and pollen data from HP7/10 and 210/297–296 samples. The top of the section (0 m) is at 101.467 m NN (= above sea level). The height between 570 and 455 cm in the ChRM-direction column is the normal intervening zone within the Blake Event (170–770 cm). The black dots of the ChRM-directions refer to first quality data points, and the open circles are of secondary quality (see SM for details). For description of units 1 to 19 and background information on the methods see SM. The part for which no palaeomagnetic samples are available (Pollen Zone I and part of Zone II) has an estimated duration of maximally 300 yr. For duration of pollen zones and sedimentation rates see Table 1. The main archaeological find horizon is situated within unit 8.

The basin (51°19′28″ N, 11°53′56″ E) developed after the deposition of a diamicton (unit 1 in Fig. 2), a till which can be up to 10 m thick in the NN2 area. The infill of the basin starts with loamy and sandy deposits that mainly consist of reworked diamicton material (unit 2 in Fig. 2), overlain by well-sorted silt loams, 6 to 8 m thick (units 3–19, Fig. 2), mostly deposited during the last interglacial. The main archaeological find horizon (within unit 8) is situated in the middle part of these silt loams (Fig. 1). The basin infill is overlain by approximately 6 m of last glacial loess.

The infill, its genesis, and the artifact-bearing deposits in particular were meticulously documented in a large number of sections throughout the excavation. A key section here is *Hauptprofil* (HP) 7 (Fig. 2), cutting through the deep part of the infill (see Fig. 1). This section was sampled for a wide range of dating techniques and palaeo-environmental studies, which include sedimentological and micromorphological studies (see Supplementary material (SM)). Further, pollen, macrobotanical remains and molluscs (for environmental reconstruction and multiple amino acid racemisation analysis) were collected. Finally, a high-resolution set of palaeomagnetic samples was acquired. It is important to note that most samples were collected from the very same part of the HP7 section, enabling a direct comparison of the results on a 5-cm stratigraphic sampling interval over the entire sequence (see SM).

All data indicate a geologically rapid infilling of a shallow basin. Micromorphological studies (see SM) show that sedimentation was a nearly continuous process, without pronounced soil formation in periods of non-deposition. Calcareous silt loams dominate the infill; these were deposited by overland flow in a very calm sedimentary setting in placid water, with only very short (~<1 decade) interruptions during which the depression fell dry. Because of the high sedimentation rate, traces of bioturbation and soil formation are virtually absent throughout the sequence, apart from some gypsum formation in the top of the sequence and occasionally occurring gley phenomena. In the upper part of the infill (the top 50 cm of the interglacial sediments), the sedimentation rate decreases.

The age of this interglacial succession is constrained by the underlying diamicton, a till of late Saalian/Drenthe age (Eissmann, 2002), and by the overlying Weichselian gravel and loessic deposits. Multiple amino acid racemisation analysis of a large series of *Bithynia tentaculata* opercula (Penkman et al., 2008) from the HP7 sequence (see SM) suggests that the deposits are contemporaneous with those at the Eemian type locality at Amersfoort (The Netherlands) (Zagwijn, 1961) and last interglacial occurrences in the United Kingdom (see also SM). Additional confirmation of the last interglacial age is provided by thermoluminescence (TL) dating of five heated flint artifacts from the archaeological level, which yielded 126 ± 6 ka as the weighted mean age estimate (see SM). Pollen studies (see SM) also demonstrate a last interglacial age for the sequence. Pollen samples were taken at sections HP7 and neighboring HP10 (cf. Figure 1, Figure 2). Unit 2, the reworked diamicton, contained pollen derived from the lignite deposits only, whereas the overlying silt loam (unit 3) was deposited in an environment with sparse vegetation at most, possibly reflecting a cold period (see SM). There is a good pollen record from unit 4 onward. Pollen is well-preserved, and the data show an interglacial succession that is typical of the Eemian interglacial in northern Europe (Turner, 2000, Zagwijn, 1961). This pollen succession starts with Pollen Zone I and ends with Zone VI/VII (*sensu* Menke and Tynni, 1984) at the top of the profile.

## The palaeomagnetic signal

A total of 184 palaeomagnetic samples were collected from the NN2 exposures: 159 samples were taken from HP7 and 25 from a section nearby, in excavation square 210/296–297. Drill cores of sufficient length were cut into two specimens and demagnetised using both alternating fields (AF) and thermal demagnetisation, resulting in a total of 216 demagnetised samples. A small part of the base of the HP7 section previously sampled for other methods became inaccessible for palaeomagnetic studies due to increased security restrictions within the quarry. Based on the pollen data, this 80 cm part represents a period of 300 yr at most (see also Fig. 2 and Table 1).

**Table 1:** Sedimentation rate in cm yr^− 1^ for NN2 HP 7/10 for individual pollen zones, based on sediment thickness at NN2 (see Fig. 2) and duration of the Eemian pollen zones as counted at the Bispingen site (Müller, 1974). To calculate the time represented by the sediments containing the Blake Event *below* Pollen Zone I (units 3 and top of 2 in Fig. 2), we used a conservative sedimentation rate of 0.2 cm yr^− 1^.

Pollen zone (Menke and Tynni, 1984)	Duration (in years) (Müller, 1974)	Sediment thickness in cm NN2 (see Fig. 2)	Sedimentation rate (cm yr^− 1^) NN2 HP7/10
I	~ 100	30	0.33
II	~ 200	90	0.45
III	~ 450	50	0.11
IVa	~ 1200	130	0.11
IVb	1000–1200	240	0.24–0.20

The samples were stepwise demagnetised progressively in AF up to 100 mT (n = 122), or thermally up to 600°C (n = 42). A selected sample set was first heated to 205°C followed by alternating field demagnetisation (to 100 mT, n = 52) to achieve optimal resolution (for the entire palaeomagnetic procedures, see SM). Typical demagnetisation diagrams (Zijderveld, 1967) are shown in Figure S7 of the SM. In the low-temperature or field range a present-day field overprint is observed, presumably of viscous origin. The characteristic remanent magnetisation (ChRM) is resolved after demagnetisation at temperatures > 200°C or alternating fields > 15–20 mT. As expected, it shows directional scatter because of secular variation of the Earth's magnetic field. A stratigraphically coherent zone (7.70–1.70 m) in the lower part of the interglacial sequence shows the clearly deviating directions that we associate with the Blake Event (Smith and Foster, 1969). In the samples with excursional directions, the overprint is always large. This is the result of a weak field during the excursion followed by a stronger field after return to stable normal polarity conditions. On top of that, a CRM overprint from greigite (NN2 is in a fresh-water setting) is acquired in the stronger post-excursional field (see SM for further details). This interferes with a clear-cut determination of the ChRM in those samples; their directions are characterized by slightly larger mean-angular deviations. The distinction between first and second quality data points (Fig. 2) is based on visual inspection of the Zijderveld diagrams. When a stable endpoint direction is observed the quality is labeled 1 (e.g., Neu117 in Fig. S7 of the SM). When curved endpoint trajectories or the directions not trending to the origin (but with GRM acquisition excluded, see SM) are noticed, quality is obviously lower and labeled as second. The distinction between the two categories is sometimes subtle given the large overprints present.

To discriminate between regular secular variation and excursional or transitional directions, we applied the variable cut-off procedure (Vandamme, 1994) that considers directional outliers as excursional (see SM, Fig. S8). First quality excursional directions (i.e., the Vandamme outliers) are restricted to the stratigraphic zone 7.70–1.70 m. Below 7.70 m only first quality data points are present, yielding a firm basis for the lower boundary of the Blake Event. In the uppermost part of the sequence, above 1.70 m, all first quality data points are normal. The ChRM directions are likely to represent the field during sedimentation; i.e., the extent to which delayed acquisition of the natural remanent magnetization may have been possible is limited, given the high sedimentation rates (Table 1). Also soil formation during deposition of the NN2 sequence is virtually absent yielding a sequence that can be considered as continuous (see SM for details).

**Figure 3:**
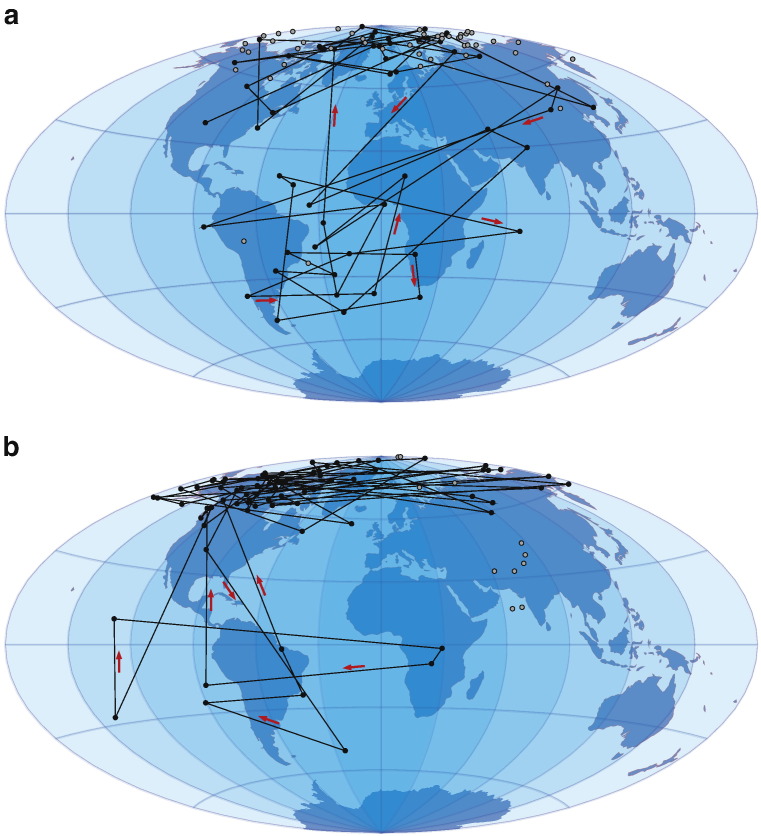
Plot of the VGP path during the Blake Event recorded in the Neumark Nord 2 basin. Panel b represents the base of the section (875 to 477 cm in Fig. 2) with a rapid (at 770 cm) first transitional phase of the record, similar to the record found by Tric et al. (1991), ending with a rebound phase, the intervening normal polarity part of the Blake Event. Panel a represents the top of the section (472 to 0 cm) with the second VGP swing and post-Blake normal NRM directions. Direction of VGP movement is indicated by the arrows. Gray dots are excluded from the VGP path.

The behaviour of the magnetic field during the Blake Event (Smith and Foster, 1969) has been described by several authors (Fang et al., 1997, Laj and Channell, 2007, Reinders and Hambach, 1995, Tric et al., 1991). It is considered a ‘double event’ with two reversed intervals, intervened by a short normal period of about 1000 yr. The ChRMs versus stratigraphic level plot (Fig. 2) as well as the plot of directions calculated as Virtual Geomagnetic Poles (VGP) (Figs. 3a and b) show this magnetic field behaviour. It appears that the entire Blake Event is recorded at the Neumark Nord 2 site.

NN2 yields the longest well-documented record of the Blake Event in a continental setting with a high sedimentation rate (see SM for more details). Moreover it is well positioned within a high-resolution pollen sequence, which allows us to set constraints on its duration, enabling further research into the understanding of geodynamo processes that generate excursions like the Blake Event (Roberts, 2008). In the NN2 pollen sequence, the base of the Blake Event is situated in the unit 2 deposits (see SM) overlying the diamicton, more specifically at 0.87 m below the deposits of Pollen Zone I (*sensu* Menke and Tynni, 1984). The natural remanent magnetization (NRM) resumes normal polarity again within Pollen Zone IVb, in the *Corylus* phase of the interglacial. Notably, neither the beginning nor the end coincides with a vegetational or a sedimentary break in the sequence. Our record resembles that described by Tric et al. (1991), in that the lower excursion lasts for a shorter time than the upper one. Also, the recovery to post-Blake normal field directions is fairly gradual. The VGPs in Tric et al.'s record extend to almost fully reversed while those in our record are more ‘intermediate’, possibly related to the large overprint in the Neumark samples.

The duration of the Eemian interglacial in northern Europe is well constrained to approximately 11,000 yr by lamination counting at the Bispingen site in northern Germany (Müller, 1974), with studies from other Eemian locations yielding comparable results (Turner, 2002). At these locations, the duration of the various vegetation zones (I–IVb) has been counted for the earlier parts of the Eemian of relevance here, i.e. the pre-temperate and temperate substages (SM). Using this robust “floating” lamination chronology (Turner, 2002) we can assign sedimentation rates to the sediment units of Figure 2 (see Table 1). We conclude that the best estimate for the duration of the Blake Event is 3400 ± 350 yr. Our calculation supports the “short chronologies” in the duration estimates, which vary from 2.8 to 8.6 ka (Tric et al., 1991, Zhu et al., 1994, Fang et al., 1997, Thouveny et al., 2004).

## Discussion

**Figure 4:**
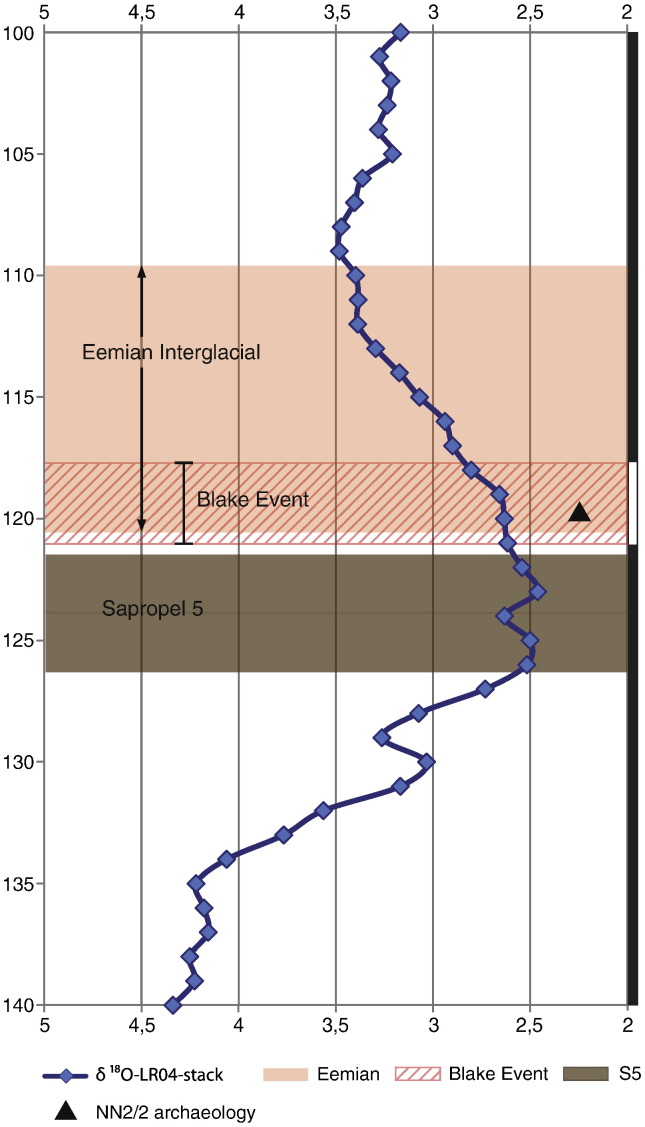
Stacked δ^18^O-LR04 record (Lisiecki and Raymo, 2005) from 140 to 100 ka, using the Lourens' (2004) time scale, with the positions of sapropel 5, the Blake Event, and the (central and northwestern European) Eemian interglacial. The summary geomagnetic polarity column is depicted farthest right. The position of the NN2/2 archaeological find-horizon is indicated as well.

The NN2 record situates the Blake Event at the very end of the Saalian and in the first 3000 yr of the terrestrial Eemian of northwestern and central Europe, making it a very good marker for the much debated Middle-Upper Pleistocene boundary (Gibbard, 2003). In the terrestrial realm, using the Blake Event palaeomagnetic signal we can start large-scale comparisons of plant and animal communities within very fine time equivalent units. The NN2 data also allow us to make the first direct correlation between the terrestrial Eemian interglacial stage with Marine Isotope Stage (MIS) stratigraphy, which has been the subject of much debate in recent years (Sánchez-Goñi et al., 1999, Shackleton et al., 2003). As in several marine cores, in cores from the Mediterranean Sea (Tucholka et al., 1987, Tric et al., 1991), the Blake Event is recorded within MIS 5e (Tucholka et al., 1987, Langereis et al., 1997, Laj and Channell, 2007) with the Blake Event beginning just above sapropel S5. For instance, in the MD84627 Blake record of Tric et al. (1991), the Blake Event starts ~ 400 yr after the deposition of sapropel S5 (see SM for discussion of possible delayed NRM acquisition). These data allow us to unambiguously correlate the NN2 data to the benthic δ^18^O curve, as visualized in Figure 4, which yields the first direct correlation of a terrestrial Eemian climate sequence to the marine realm.

There are various age estimates for the S5 sapropel, with Lourens' (2004) midpoint age estimate of 124 ka generally accepted. Tucholka et al. (1987) showed a ~ 5 ka duration of S5 in the Tric et al.'s record, which gives the top of S5 an age of ~ 121.5 ka. The Blake Event started at 121.1 ± 0.5 ka under the proviso that the excursional behaviour is spatially synchronous within uncertainty (we compare an eastern Mediterranean with a central European record). In that age model the Eemian as recorded at NN2 would start at ~ 120.65 ka and last until ~ 109.65 ka.

The age of the Blake Event, and of the various Eemian vegetation zones at NN2 is dependent on the age estimate of sapropel S5; it will change along with possible future changes in the age estimate of that sapropel. The *position* of the terrestrial Eemian as recorded at NN2 within the MIS record is, however, fixed. Therefore, the NN2 data allow a firm correlation of a high-resolution terrestrial environmental sequence to the marine record. This will serve as a point of departure for future studies of the vegetation succession, climate change and palaeomagnetic data in a terrestrial setting, and their much debated relationships to contemporary events reflected in the deep sea record (Kukla, 2000) (Fig. 4).

The best data for correlation between the two realms thus far has come from a study of terrestrial pollen from marine sediments studied in core MD95-2042 off the coast of the Iberian Peninsula (Sánchez-Goñi et al., 1999, Shackleton et al., 2003). In contrast, the NN2 data allow us to correlate a high-resolution terrestrial record to the marine record by means of the palaeomagnetic signal of the Blake Event. Our positioning of the Eemian interglacial as recorded at NN2 in the LR04-stack record (Lisiecki and Raymo, 2005) shows that the beginning of the Eemian interglacial was significantly later than the attainment of the MIS 5e plateau in benthic δ^18^O (Shackleton et al., 2003). This makes for an interesting difference with the Iberian offshore record, where the beginning of the Eemian as delimited in core MD95-2042 corresponds to the lightest isotope values of MIS 5e (Shackleton et al., 2002). Our data shows that the Eemian of central and northwestern Europe starts with a return to the heavier values toward the MIS 5e/5d transition, i.e. an estimated 5000 yr later than in the south. The estimates for the end of the Eemian interglacial in both areas are remarkably similar, however (Sánchez-Goñi et al., 1999, Shackleton et al., 2002).

Independent of these time scales, the beginning of the Eemian interglacial as documented at NN2 occurs not simply after the major ice sheets had melted, but considerably later, when sea levels had already began to drop and substantial continental ice was once again accumulating (Fig. 4). These findings may have major implications for views on the relationships between events recorded in the marine record and in the terrestrial realm, and might require a revision to the current framework of understanding regarding the timing of the Eemian of central Europe relative to MIS 5e (Tzedakis et al., 2009).
