# Supplementary material for: Direct terrestrial–marine correlation demonstrates surprisingly late onset of the last interglacial in central Europe
Source: Quat Res. 2011 Jan;75(1):213–8. doi: 10.1016/j.yqres.2010.11.003 (PMC4600610; doi:10.1016/j.yqres.2010.11.003)
Supplement: Supplementary file 1 — Supplementary material [file mmc1.doc]

Supplementary Material Sier et al.

Stratigraphical and palaeoenvironmental context of the Neumark-Nord 2 archaeological site (Saxony-Anhalt, Germany)

**Introduction**

The eastern part of Germany has played a key role in studies of the glaciations in Europe. There is ample evidence for two major glacier advances with many additional minor oscillations of Elsterian and Saalian age. A sequence of more than 50 horizons and complexes of glacial and periglacial facies has been identified in this geologically rich area (Eissmann, 2002). Maps of the palaeogeography of the region during the periods of the major European continental glaciations belong to the stock and trade of textbooks on the Quaternary. Quaternary deposits were and still are well exposed in the large-scale open-cast lignite mines in the area. Here we report on the results of interdisciplinary studies of the geology and archaeology of the Neumark-Nord exposures, in a lignite quarry situated approximately 10 km southwest of Halle, in the Geisel valley.

The Geisel valley area is well-known for its Eocene fossils, but intensive explorations of the Pleistocene exposures in the lignite quarries during the last three decades have uncovered large amounts of Middle and Late Pleistocene fossils, often in association with traces of hominin activities. At Neumark-Nord these have been retrieved from the infill of three shallow basins, which formed as a result of isostatic movements induced by lignite diapirism (Mania et al., 1990; Thomae, 2003). The well-known lake basin of Neumark-Nord 1 was studied in the 1980s and 1990s by a team under the direction of Dietrich Mania (Mania and Mania, 2008; Mania et al., 1990). They uncovered remarkable scatters of bones and partially articulated animal carcasses on the shores of a former lake, occasionally associated with flint artifacts. Deeper parts of the lake yielded c. 150 (more or less complete) fallow deer skeletons as well the remains of >26 straight tusked elephants.

During the fieldwork in the lignite quarry in 1996, Mania discovered a second basin, Neumark-Nord 2 (NN2), a few hundred meters to the north-east of Neumark-Nord 1. In 2003 a third basin was identified, the oldest of the three (NN3) (Laurat and Brühl, 2006). The *Landesamt für Denkmalpflege und Archäologie Sachsen-Anhalt* started formal excavations at NN2 in 2003. Directed by T. Laurat and E. Brühl these excavations focused on an early Weichselian find level in the upper part of the basin’s infill (NN2/0). During the construction of a geological trench the interglacial find horizon discussed in this paper was discovered in the silty infill of the basin. Since 2004, the NN2/2 find levels have been under excavation, from 2006 onward in a joint project of the *Landesamt für Denkmalpflege und Archäologie Sachsen-Anhalt*, the *Römisch-Germanisches Zentralmuseum* at Mainz (Germany) and the Faculty of Archaeology of Leiden University (The Netherlands). Weather conditions permitting, an excavation crew was in the field all year round, until the end of August 2008. By then the water level in the flooding former quarry which is to be turned into a recreation resort, reached the NN2/2 find levels. In total, an area of 433 m^2^ was excavated, yielding a rich archaeological assemblage, containing c. 20,000 Middle Palaeolithic flint artifacts and approximately 120,000 faunal remains representing a warm temperate fauna which includes straight tusked elephant, rhinoceros, bovids, equids, deer, bear, small carnivores and the pond tortoise *Emys orbicularis*.

**Description of Profile HP7 (H.J. Mücher, N. Hesse, L. Kindler & W. Roebroeks)**

A key section in the geological studies of the NN2 exposures is *Hauptprofil* (HP) 7, one of the many sections described inside and around the excavated site. This section was sampled for a wide range of dating and palaeo-environmental techniques. All samples were collected from the very same part of the section, enabling a direct comparison of the results on a 5 cm stratigraphic sampling interval over the entire sequence, up tot 600 cm below the top of the section. Stratigraphically lower samples were taken from neighbouring sections within the excavation area, excavation square section 210/296-297 for palaeomagnetic samples and for palynological sampling at HP10, 8 m from HP 7, stratigraphically partly overlapping and underlying the sampled base of HP7. The *Pinus-Betula* phase in this section revealed such a good overlap with the HP7 section that the results of both sections may be presented as one single pollen diagram (see Bakels, Supplementary Material (SM)).

At the Neumark-Nord 2 site, the basin infill is underlain by a late Saalian (Drenthian) sandy-gravelly diamicton (Unit 1 in Figure 2) and overlain by ca. 6 metres of last glacial (Weichselian) loess. The basin infill starts with sediments reworked from the diamicton (unit 2 in Fig 2), overlain by a series of mostly well-drained, moist, calcareous silt loams, which has been divided into several sublayers (units 3-19), described below, from the bottom of the sequence going towards the top of the exposed sediments at HP7, and using FAO guidelines for soil profile description (FAO, 1977). At the Neumark-Nord 2 site, data on the sediments underlying the silt loams (i.e. sedimentary units 1-2, total thickness c. 300 cm) were mainly obtained by augering. The numbering scheme adopted below refers to the layer numbers in Figure 2 of the paper.

1 Sandy loamy diamicton, with gravel and well-rounded quartz and prophyr particles, dark grayish-brown in colour, up to 150 cm thick, with a more yellowish-brown top (at HP7/10 only observed through augering).

2 Series of loamy and sandy deposits with gravel particles, occasionally laminated sand layers, up to 175 cm thick (at HP7/10 only observed through augering) interpreted as reworked from the underlying diamicton.

3 Light-brown to brown (7.5 YR 5/4-6/4) silt loam, calcareous, 50 cm thick, structureless, very friable, no cutans, no cementation, no pores, no stone and mineral fragments, no mineral nodules, nature with boundary below not observed.

4 Olive black (5Y 3/1) silty clay, moist, calcareous, 0-50 cm thick, structureless, abrupt and smooth boundary with horizon below.

5 Dark gray (5Y 4/1), moist, calcareous structureless silt loam, c. 70 cm thick, friable to firm, few distinct sharp brown (7.5YR 5/8) iron mottles; vertical reddish yellow (7.5 YR 6/6) infillings of cracks with silt loam and bands of silt; very few, very fine, discontinuous, in ped tubular, channels; very few very small shell fragments; abrupt and smooth boundary with horizon below. One undisturbed thin section sample: M3lo-1.

6 Pinkish gray to brown (7.5YR 6/2-5/2, moist), calcareous, structureless, silt loam, 35 cm

thick, very friable; very few, fine, faint, pink (5YR 7/4) iron mottles; darker rectangular and

rounded lumps of soil material with lignite, diameter resp. 4x10 and 2x2 cm; lower part

contains bone fragments, flint artefacts and mollusc shells; abrupt boundary with horizon

below. One undisturbed thin section sample: M3lo-2.

7 Yellowish brown (10YR 5/4), very fine laminated, sub-horizontal, moist, calcareous silt loam, 65 cm thick, very friable to friable, and pinkish gray (7.5YR 6/2) silt on top (2 cm thick); in the bottom, 10 cm thick, a very dark gray (5YR 3/1) calcareous organic band; very few fine iron mottles, reddish yellow (5YR 6/6) with distinct sharp boundaries; with very prominent laminae; one wedge infilling, 11 cm deep and 2 cm wide; abrupt and smooth boundary with horizon below. With bone fragments and flint artefacts. Three undisturbed thin section samples: M3lo-5 in top organic layer; M3lo-4 in very fine laminated silt loam; M3lo-3 in bottom organic layer (see Figures S2 and S3 for a close-up of the thin section).

8 Yellowish brown (10YR 5/4) moist, calcareous, structureless, silt loam, 50 cm thick, very friable, very few reddish yellow (5YR 6/8) medium iron mottles with distinct and sharp boundaries; abrupt and smooth boundary with horizon below – main find level, numerous bone fragments and flint artefacts. One undisturbed thin section sample M3lo-6.

9 Brown (10YR 5/3), calcareous, structureless, silt loam; 80 cm thick, friable to firm; very few, small to large, hard, irregular, flat, black, calcareous nodules; boundary with horizon below abrupt and smooth. Few bone fragments and flint artefacts. One undisturbed sample: M3u-1 (see Figure S4 for a close up of the thin section).

10 Yellowish brown (10YR 5/4), calcareous, medium to coarse, subangular blocky, silt loam, 50 cm thick, firm; very few gravel-sized quartz, fresh, angular and rounded; very few bones and lithics ; abrupt and smooth boundary with horizon below. One undisturbed thin section sample: M3u-2.

11 Very pale brown (10YR 7/3) calcareous, structureless, silt loam, 30 cm thick, very friable; very few, gravel-sized quartz, fresh, angular and rounded; few artefacts and bones; abruptand smooth boundary with horizon below. One undisturbed thin section sample: M3u-3.

12 Pale brown (10YR 6/3) calcareous, structureless, silt loam; 20 cm thick; very friable; few, very fresh, rounded and angular, rock fragments; very few artefacts and bones ; abrupt and smooth boundary with horizon below. One undisturbed thin section sample: M3u-4.

13 Light gray to light brownish gray (2.5Y 7/2-2.5Y 6/2), calcareous, structureless, silt loam, 20 cm thick, friable; many fine prominent, sharp, reddish yellow (7.5YR 7/8) iron mottles; very few, very fine, discontinuous, oblique, inped, tubular, simple pores; very few artefacts and bones ; very few, very fine, black roots; abrupt and smooth boundary with horizon below. One undisturbed thin section sample: M3u-5 in calcium carbonate band.

14 Light gray (10YR 7/2), calcareous, structureless, silt loam, 80 cm thick, friable; very few, gravel-sized (< 0.5 cm in diameter), rounded fresh quartz; common, medium to coarse, prominent, sharp, reddish yellow (7,5YR 7/6) iron mottles; they occur also along vertical cracks; in sub-horizontal bands at 45 and 65 cm from horizon bottom, very few, large (up to 2 cm in diameter), hard, and rounded, white calcium carbonate nodules occur; in the top a gypsum layer; very few artefacts and bones ; very few pedotubules with pinkish white (7.5YR 8/4), loose infilling of silt; abrupt and smooth boundary with horizon below. One undisturbed thin section sample: M3u-6 in upper gypsum band.

15 Dark reddish gray (5YR 4/2), lower part structureless, silty clay loam, 20 cm thick, in upward direction platy silty clay loam, friable to firm; organic material increases towards the top of the horizon, the organic matter is laminated, with modern roots; common coarse prominent, sharp, brown (10YR 4/2) iron mottles; sharp and smooth boundary with horizon below. One undisturbed thin section sample: M3u-7.

16 Pinkish white (7.5YR 8/2) calcareous, structureless, silt loam, 5-10 cm thick, (“Seekreide”) firm; it is covered by a 2 cm thick, laminated, dark reddish brown (5YR 3/2) organic material; abundant molluscs and fragments of it; few recent roots; abrupt and smooth boundary with horizon below. One undisturbed thin section sample M3u-8.

17 Olive brown (2.5Y 4/4) calcareous, structureless, silt loam, 10 cm thick, friable to firm; few, fine to medium, random, inped, tubular pores (root channels?); very few bone fragments and flint artefacts and few molluscs fragments. Abrupt and smooth boundary with horizon below. One undisturbed thin section sample: M3u-9.

18 Light yellow brown (10YR 6/4) calcareous, structureless, silt loam, 30 cm thick, very friable; many medium, distinct and sharp, strong brown (7.5YR 5/8) iron mottles; few shell fragments; Clear and smooth boundary with horizon below. One undisturbed thin section sample: M3u-10.

19 Yellow (10YR 7/8), moist, calcareous, structureless, loamy sand, more than 70 cm thick

(top not observed) with locally intercalated sand layers; few medium, distinct and sharp,

Strong brown (7.5YR 5/8); consistence: loose to soft. Abrupt and smooth boundary with

horizon below.

**Micromorphological study of the thin sections: summary of results (H.J. Mücher)**

# The soil formation in the silt loam sediment is very weak, consisting of incipient gleysols and cambisols (FAO-Unesco, 1988). The sedimentation by overland flow was an almost continuous process. Very weakly pronounced pedogenesis is restricted to very local occurrences of platy and blocky structures, crust formation, and in addition Ca, Fe, and Mn coatings and hypocoatings, crystals of gypsum and carbonate. The presence of these pedofeatures (Stoops, 2003) was inferred on basis of a macroscopic study of the deposits, and confirmed by micromorphological analysis of thin sections, 9 x 6 cm and 20 µm thick.

# The mainly calcareous sediment was deposited by overland flow, mainly by after flow and less by rain wash (transport by splash cannot be excluded). Crusts and clay lamina suggest a very short interruption in the sedimentation process, with respectively crust formation (Figure S2: M3lo-3, plain polarized light [PPL] and Figure S3, the same object but in crossed polarized light [XPL]) and sedimentation in still waters. In addition, during these very short stable periods micro-rills were formed in the temporary soil surface Figure S4: M3u-1 in PPL).

Physical modifications after deposition are rare, examples are cracks, micro-rills followed by infillings, and wavy deformation of lamina. Indications for frost action have not been observed.

Soil formation phenomena are rare, only incipient soil formation was observed with development of platy structures in thin section M3u-4, and of blocky peds in M3u-2. Recrystallization of carbonates creating carbonate coatings, carbonate hypocoatings and carbonate nodules, and crystallization of gypsum in dry conditions occurred only in the upper part of the profile. These dry conditions were often followed by a rising groundwater table resulting in the formation of gley phenomena such as ferric and manganiferous nodules, Fe-Mn coatings and hypocoatings. The gley phenomena often cover the gypsum formation, indicating that the hydromorphic features developed after the gypsum crystallizations.

**Palynology (C. C. Bakels)**

During the analysis of section HP7 the pollen detected in its lowest part, 595 cm from the top of HP7, was found to represent a *Pinus-Betula* phase with a dominance of *Pinus*. To study the very oldest phase of the vegetation succession another excavation section at 8 m from HP7, HP10, was sampled which stratigraphically partly overlaps and underlies the sampled base of HP7. The *Pinus-Betula* phase in this section revealed such a good overlap with the HP7 section that the results of both sections may be presented as one single pollen diagram.

The sediments were treated according to standard practice with KOH, HCl, heavy liquid separation (s.g. 2.0) and acetolysis. Before treatment tablets containing a known number of *Lycopodium* spores were added, according to Stockmarr’s method (Stockmarr, 1971). This procedure enables the calculation of pollen concentrations. The diagram presented here is, however, not a concentration diagram but a conventional percentage diagram, based on a pollen sum consisting of trees and herbs from non-aquatic environments. For this sum at least 300 pollen and spores were counted; only in pollen zone II and the centre of pollen zone IVb this number could not be reached because of a low pollen density. To avoid an unwieldy graph those taxa which were found not too often are presented in Table S1.

The diamicton and reworked diamicton of the units 1 and 2 (see main text Figure 2) contained only pollen and spores which were obviously derived from much older sediments, possibly from the lignite. Unit 3 did not contain pollen at all. This layer consists of a light-brown silt loam, possibly reworked loess, deposited in an environment with sparse vegetation at most. If any pollen was released at that time it has not been preserved. It is only after this cold period that pollen is present again, trapped in the wet silty clays of unit 4. From this unit onwards pollen and spores reflect the actual vegetation. Preservation was good. Some fossil palynomorphs were encountered and provide a kind of ‘noise’ amidst the pollen and spores presumably shed by the plants living during the time of the deposition of the sediments, but these fossils were of quite different appearance and could readily be discerned from younger material.

The pollen and spores represent the vegetation of an interglacial and the presence of *Ilex* and *Hedera* implies that this interglacial was oceanic in character. This, together with arguments brought forward by other methods reported in this paper, concurs with the interglacial being the Eemian. The local pollen zones fit the zonation proposed by Menke and Tynni (Menke and Tynni, 1984) for Northern Germany very well and their zone numbers are used to describe the diagram further on (Turner, 2000).

The sequence starts with a phase dominated by *Betula* and herbs (zone I). Part of the *Betula* pollen in the two lowest spectra is of the *Betula nana* type. The *Betula* phase is followed by a *Pinus* phase with some *Betula* (zone II) and this again by a *Pinus-Quercus* phase (zone III). The main part of the diagram belongs to zone IV, in which firstly *Quercus* and *Ulmus*, then *Quercus* and *Corylus* and in the end *Corylus* alone dominate the pollen assemblage. Zones III and IV concord with the early-temperate phase of Turner and West (Turner and West, 1968) and the Eemian pollen zones E3 and E4 as defined in Turner (Turner, 2002). The next zone, zone V, dominated by *Carpinus*, is not present in its usual length, because of an apparent hiatus in the deposits at the very top of HP7. This hiatus is to be placed at the end of stratigraphical unit 17 (see above). The next pollen zone shows a dominance of *Pinus*.

During the zones I-IV herb pollen is present in considerable numbers. In the case of zone IV this is remarkable. Halfway zone IVb, the *Corylus* phase, the vegetation appears almost treeless. The peak in *Pinus* pollen is attributable to the absence of other trees. The NN2 basin is small, and the open aspect of the vegetation is interpreted as reflecting mostly very local conditions (Tauber, 1965; Tauber, 1967). It is probable that large mammals came here to drink, and to graze and browse along its shores. The high percentages of *Polygonum aviculare* pollen indicate the presence of much trodden areas. It is even possible that some of the herb pollen has its origin in animal dung. The presence of *Sordaria* spores in some of the samples confirms the presence of dung. During the period of *Carpinus* dominance the records lack high percentages of herbs. The vegetation must have been true woodland at that time.

Table S1: Pollen and spores not given in the diagram of figure S5, arranged according to their frequency in the zones and subzones. Black squares indicate presence of taxon.

| Neumark-Nord 2 HP7/HP10 |  |  |  |  |  |  |  |  |  |  |  |
| --- | --- | --- | --- | --- | --- | --- | --- | --- | --- | --- | --- |
|  |  |  |  |  |  |  |  |  |  |  |  |
| Zone | VII | V | IVb3 | IVb2 | IVb1 | IVa2 | IVa1 | III | II | I |  |
| **Trees, shrubs, lianas** |  |  |  |  |  |  |  |  |  |  |  |
| *Alnus* | ■ | ■ | ■ | ■ | ■ | ■ | ■ | ■ | ■ | - | 9 |
| *Tilia* | - | ■ | ■ | ■ | ■ | ■ | ■ | ■ | - | - | 7 |
| *Salix* | - | - | ■ | - | ■ | ■ | ■ | ■ | ■ | ■ | 7 |
| Rosacae | - | - | ■ | ■ | ■ | ■ | - | ■ | ■ | - | 6 |
| *Ilex* | - | ■ | - | ■ | - | ■ | - | ■ | - | - | 4 |
| *Acer* | - | ■ | - | - | ■ | ■ | - | - | - | - | 3 |
| *Populus* | - | - | ■ | ■ | ■ | - | - | - | - | - | 3 |
| *Cornus sanguinea* | - | - | - | - | ■ | ■ | ■ | - | - | - | 3 |
| *Humulus/Cannabis* | - | - | - | - | ■ | ■ | - | ■ | - | - | 3 |
| *Taxus* | - | ■ | - | - | ■ | - | - | - | - | - | 2 |
| *Hedera* | - | - | - | - | - | ■ | ■ | - | - | - | 2 |
| *Ligustrum* | - | - | ■ | - | - | - | - | - | - | - | 1 |
| *Rhamnus* | - | - | - | - | ■ | - | - | - | - | - | 1 |
| *Myrica* | - | - | - | - | - | ■ | - | - | - | - | 1 |
| *Frangula* | - | - | - | - | - | ■ | - | - | - | - | 1 |
| *Prunus* | - | - | - | - | - | ■ | - | - | - | - | 1 |
| **Herbs, open space** |  |  |  |  |  |  |  |  |  |  |  |
| *Galium* type | ■ | ■ | ■ | ■ | ■ | ■ | ■ | ■ | - | ■ | 9 |
| *Filipendula* | ■ | ■ | ■ | ■ | ■ | ■ | ■ | - | - | ■ | 8 |
| *Ranunculus acris* type | - | ■ | ■ | ■ | ■ | ■ | ■ | ■ | - | ■ | 8 |
| Ericales | - | ■ | ■ | ■ | ■ | ■ | ■ | ■ | - | - | 7 |
| Apiaceae | ■ | - | ■ | ■ | ■ | ■ | ■ | ■ | - | - | 7 |
| Brassicaceae | - | - | ■ | - | - | ■ | ■ | ■ | ■ | ■ | 6 |
| *Rumex acetosa* type | - | ■ | ■ | - | - | ■ | ■ | ■ | ■ | - | 6 |
| *Caltha* | - | - | - | - | - | ■ | ■ | ■ | ■ | ■ | 5 |
| *Triglochin* | - | ■ | ■ | - | ■ | ■ | - | - | - | ■ | 5 |
| *Plantago coronopus* | - | - | ■ | ■ | - | ■ | - | ■ | ■ | - | 5 |
| *Trifolium* type | - | - | ■ | - | ■ | ■ | - | ■ | - | - | 4 |
| *Campanula* | - | - | - | - | ■ | ■ | - | ■ | ■ | - | 4 |
| *Thalictrum* | - | - | - | - | - | ■ | - | ■ | ■ | ■ | 4 |
| *Plantago major/media* | ■ | - | - | - | - | ■ | - | ■ | - | ■ | 4 |
| *Spergularia* | - | - | ■ | - | ■ | ■ | - | - | - | - | 3 |
| *Centaurea jacea* type | - | - | - | - | ■ | - | - | - | ■ | ■ | 3 |
| *Veronica* | - | - | - | - | ■ | ■ | ■ | - | - | - | 3 |
| *Equisetum* | ■ | - | ■ | - | - | - | - | ■ | - | - | 3 |
| *Geranium* | - | - | ■ | - | ■ | - | - | - | - | - | 2 |
| *Stachys* type | - | - | - | - | ■ | ■ | - | - | - | - | 2 |
| *Melampyrum* | - | - | - | - | ■ | - | - | ■ | - | - | 2 |
| *Scrophularia* type | - | ■ | - | - | ■ | - | - | - | - | - | 2 |
| *Polygonum bistorta* type | - | - | - | - | - | - | - | ■ | ■ | - | 2 |
| *Mentha* type | - | - | ■ | - | - | ■ | - | - | - | - | 2 |
| *Jasione montana* type | - | - | ■ | ■ | - | - | - | - | - | - | 2 |
| *Papaver rhoeas* type | - | - | - | ■ | - | ■ | - | - | - | - | 2 |
| *Helianthemum nummularium* type | - | - | - | ■ | - | ■ | - | - | - | - | 2 |
| *Sanguisorba officinalis* | - | - | - | ■ | ■ | - | - | - | - | - | 2 |
| *Lysimachia vulgaris* type | - | - | - | ■ | - | ■ | - | - | - | - | 2 |
| *Saxifraga aizoides* type | - | - | - | - | ■ | ■ | - | - | - | - | 2 |
| *Vicia type* | - | - | ■ | - | - | - | - | - | - | - | 1 |
| *Chamaenerion* | - | - | - | - | - | - | - | ■ | - | - | 1 |
| *Prunella type* | - | - | - | - | ■ | - | - | - | - | - | 1 |
| *Polypodium* | ■ | - | - | - | - | - | - | - | - | - | 1 |
| *Onobrychis* | - | - | - | - | - | ■ | - | - | - | - | 1 |
| *Parietaria* | - | - | - | - | - | ■ | - | - | - | - | 1 |
| *Solanum dulcamara* | - | - | ■ | - | - | - | - | - | - | - | 1 |
| *Teucrium chamaedrys* type | - | - | - | - | - | - | - | - | - | ■ | 1 |
| *Gentianella germanica* type | - | - | - | ■ | - | - | - | - | - | - | 1 |
| *Potentilla* | - | - | - | ■ | - | - | - | - | - | - | 1 |
| *Fumana procumbens type* | - | - | - | ■ | - | - | - | - | - | - | 1 |
| *Plantago alpina/maritima* | - | - | - | - | - | - | - | - | - | ■ | 1 |
| **Waterside and lake vegetation** |  |  |  |  |  |  |  |  |  |  |  |
| *Sparganium erectum type* | - | ■ | - | - | ■ | ■ | - | ■ | - | - | 4 |
| *Typha latifolia* | - | ■ | - | - | ■ | ■ | - | - | ■ | - | 4 |
| *Potamogeton* | - | ■ | - | - | - | - | - | ■ | ■ | - | 3 |
| *Mougeotia* | - | ■ | - | - | ■ | - | - | - | - | - | 2 |
| *Zygnema* | - | - | ■ | - | - | - | - | ■ | - | - | 2 |
| *Pediastrum* | ■ | ■ | - | - | - | - | - | - | - | - | 2 |
| *Alisma* | - | - | - | ■ | - | - | - | - | - | - | 1 |
| *Myriophyllum spicatum* | - | - | - | - | - | ■ | - | - | - | - | 1 |
| *Myriophyllum verticillatum* | - | - | - | - | - | - | - | - | ■ | - | 1 |
|  |  |  |  |  |  |  |  |  |  |  |  |
| *Sordaria* type | - | - | - | - | - | - | ■ | ■ | ■ | - | 3 |

**Amino Acid Analysis (K.E.H. Penkman)**

A new technique of amino acid analysis has been developed for geochronological purposes (Penkman, 2005; Penkman et al., 2008a; Penkman et al., 2008b; Penkman et al., 2007) combining a recent reverse-phase high-pressure liquid chromatography (RP-HPLC) method of analysis (Kaufman and Manley, 1998) with the isolation of an 'intracrystalline' fraction of amino acids by bleach treatment (Sykes et al., 1995). This combination of techniques results in the analysis of D/L values of multiple amino acids from the chemically protected protein within the biomineral, thereby enabling both smaller sample sizes and increased reliability of the analysis compared to more conventional amino acid analysis protocols. Amino acid data obtained from the intra-crystalline fraction of the calcitic *Bithynia* opercula indicate that this biomineral is a particularly robust repository for the original protein (Penkman, 2005; Penkman et al., 2008b; Penkman et al., 2009) and therefore has been targeted in this study.

Amino acid racemisation (AAR) analyses were undertaken on 42 individual *Bithynia tentaculata* opercula from Neumark-Nord 2 from HP7 (NEaar 4714-4717; 4927-4930; 5628-5650; 5687-5697). All samples were prepared using the procedures of Penkman *et al*. (Penkman et al., 2008a) to isolate the intra-crystalline protein by bleaching. Two subsamples were then taken from each shell; one fraction was directly demineralised and the free amino acids analysed (referred to as the 'Free' amino acids, FAA or F), and the second was treated to release the peptide-bound amino acids, thus yielding the 'total' amino acid concentration, referred to as the ‘Total Hydrolysable amino acid fraction (THAA or H*). Samples were analysed in duplicate by RP-HPLC. During preparative hydrolysis, both asparagine and glutamine undergo rapid irreversible deamination to aspartic acid and glutamic acid respectively (Hill, 1965). It is therefore not possible to distinguish between the acidic amino acids and their derivatives and they are reported together as Asx and Glx respectively.

The D/L ratios of aspartic acid/asparagine, glutamic acid/glutamine, serine, alanine and valine (D/L Asx, Glx, Ser, Ala, Val) as well as the [Ser]/[Ala] value are then assessed to provide an overall estimate of protein decomposition. Serine is one of the most geochemically unstable amino acids, with one of its decomposition products being alanine (Bada et al., 1978). This enables the ratio of the concentration of serine ([Ser]) to the concentration of alanine ([Ala]) to be used as a useful indication of the extent of protein decomposition (Parfitt et al., 2005). The D/L of an amino acid will increase with increasing time, whilst the [Ser]/[Ala] value will decrease. Each amino acid racemises at different rates, and therefore is useful over different timescales. The D/L of Ser is less useful as a geochronological tool for samples of this age, but is presented here as aberrant values are useful indications of contamination.

In a closed system, the amino acid ratios of the FAA and the THAA subsamples should be highly correlated, enabling the recognition of compromised samples (Preece and Penkman, 2005). The *Bithynia tentaculata* opercula from Neumark-Nord 2 show very consistent data (Table S2; Figure S6), indicating that nearly all the samples contain closed-system protein and therefore can be reliably used for age estimation. However, three opercula showed divergent within-sample behaviour: one from a depth of 395 cm (NEaar 5642), one from a depth of 445 cm (NEaar 5643) and one from a depth of 540 cm (NEaar 4927), and data from these samples should not be used.

The extent of protein decomposition in both the FAA and THAA increases with time, with increased levels of protein breakdown during warm stages and a slowing in the rates of degradation in cold stages. Over a small geographical area, it can be assumed that the integrated temperature histories are effectively the same. Given a similar temperature history, this then allows an aminostratigraphic framework for an area to be developed, plotting the FAA against the THAA data, with independent geochronology allowing these clusters to be correlated to marine oxygen isotope warm stages; such a framework has been developed for opercula from the United Kingdom (Penkman, 2005; Penkman et al., 2008b). The range of data for British sites has been compared to the Neumark-Nord 2 dataset (Fig. S5), and it is clear that the Neumark-Nord 2 samples have amino acid ratios that are similar to, but slightly higher than, those for the same species from sites correlated with the British Ipswichian (and therefore the Eemian and MIS 5e (Bowen, 1999)), including Trafalgar Square, Bobbitshole, Coston, Shropham, Cropthorne New Inn, Eckington and Tattershall Castle (Penkman et al., 2008b). The integrated temperature history for more continental sites may not be identical to that experienced by the British opercula, and therefore the overall state of degradation will be different for samples of identical age. A framework for continental Europe is under development, but, importantly, the data from opercula from the Eemian type locality at Amersfoort (The Netherlands) (Zagwijn, 1961) show similar levels of protein degradation as the Neumark samples.

Table S2: Amino acid data on opercula of *Bithynia tentaculata* from Neumark-Nord 2. Error terms represent one standard deviation (σ) about the mean (av) for the duplicate analyses for an individual sample. Each sample was bleached (b), with the free amino acid fraction signified by ‘F’ and the total hydrolysable fraction by ‘H*’.

| NEaar no. | Sample name | Asx D/L | | Glx D/L | | Ser D/L | | Ala D/L | | Val D/L | | [Ser]/[Ala] | |
| --- | --- | --- | --- | --- | --- | --- | --- | --- | --- | --- | --- | --- | --- |
|  |  | av | σ | av | σ | av | σ | av | σ | av | σ | av | σ |
| 4714bF | NN2100Bto1bF | 0.596 | 0.002 | 0.186 | 0.001 | 0.862 | 0.002 | 0.219 | 0.007 | 0.156 | 0.006 | 0.665 | 0.017 |
| 4714bH* | NN2100Bto1bH* | 0.496 | 0.001 | 0.137 | 0.004 | 0.485 | 0.004 | 0.162 | 0.005 | 0.093 | 0.000 | 0.614 | 0.016 |
| 4715bF | NN2100Bto2bF | 0.599 | 0.000 | 0.185 | 0.003 | 0.875 | 0.001 | 0.230 | 0.001 | 0.161 | 0.008 | 0.799 | 0.014 |
| 4715bH* | NN2100Bto2bH* | 0.521 | 0.002 | 0.144 | 0.002 | 0.572 | 0.004 | 0.167 | 0.004 | 0.094 | 0.002 | 0.649 | 0.004 |
| 4716bF | NN2100Bto3bF | 0.573 | 0.000 | 0.174 | 0.000 | 0.875 | 0.000 | 0.234 | 0.001 | 0.161 | 0.007 | 0.782 | 0.003 |
| 4716bH* | NN2100Bto3bH* | 0.483 | 0.002 | 0.132 | 0.000 | 0.506 | 0.002 | 0.163 | 0.000 | 0.093 | 0.001 | 0.594 | 0.000 |
| 4717bF | NN2100Bto4bF | 0.592 | 0.007 | 0.193 | 0.005 | 0.890 | 0.002 | 0.224 | 0.001 | 0.174 | 0.006 | 0.784 | 0.002 |
| 4717bH* | NN2100Bto4bH* | 0.485 | 0.002 | 0.139 | 0.001 | 0.529 | 0.000 | 0.163 | 0.004 | 0.093 | 0.000 | 0.576 | 0.004 |
| 4927bF | NN2540Bto1bF | 0.770 | 0.002 | 0.971 | 0.008 | 0.191 | 0.270 | 0.877 | 0.000 | 1.033 | 0.021 | 0.010 | 0.002 |
| 4927bH* | NN2540Bto1bH* | 0.667 | 0.003 | 0.850 | 0.006 | 0.073 | 0.103 | 0.841 | 0.006 | 0.754 | 0.005 | 0.016 | 0.011 |
| 4928bF | NN2540Bto2bF | 0.640 | 0.000 | 0.206 | 0.000 | 0.933 | 0.002 | 0.267 | 0.001 | 0.183 | 0.002 | 0.631 | 0.002 |
| 4928bH* | NN2540Bto2bH* | 0.515 | 0.003 | 0.155 | 0.000 | 0.576 | 0.000 | 0.193 | 0.002 | 0.108 | 0.001 | 0.524 | 0.003 |
| 4929bF | NN2540Bto3bF | 0.633 | 0.001 | 0.210 | 0.008 | 0.924 | 0.005 | 0.255 | 0.002 | 0.183 | 0.009 | 0.661 | 0.004 |
| 4929bH* | NN2540Bto3bH* | 0.492 | 0.002 | 0.144 | 0.000 | 0.528 | 0.007 | 0.184 | 0.000 | 0.095 | 0.001 | 0.549 | 0.003 |
| 4930bF | NN2540Bto4bF | 0.634 | 0.001 | 0.227 | 0.003 | 0.922 | 0.002 | 0.284 | 0.004 | 0.198 | 0.011 | 0.649 | 0.003 |
| 4930bH* | NN2540Bto4bH* | 0.540 | 0.001 | 0.166 | 0.001 | 0.610 | 0.002 | 0.205 | 0.002 | 0.118 | 0.002 | 0.514 | 0.000 |
| 5628bF | NN2110Bto1bF | 0.610 | 0.025 | 0.212 | 0.008 | 0.938 | 0.016 | 0.242 | 0.002 | 0.158 | 0.002 | 0.871 | 0.001 |
| 5628bH* | NN2110Bto1bH* | 0.504 | 0.013 | 0.136 | 0.001 | 0.570 | 0.005 | 0.164 | 0.005 | 0.086 | 0.002 | 0.671 | 0.001 |
| 5629bF | NN2110Bto2bF | 0.625 | 0.022 | 0.212 | 0.006 | 0.951 | 0.003 | 0.252 | 0.000 | 0.176 | 0.004 | 0.877 | 0.014 |
| 5629bH* | NN2110Bto2bH* | 0.522 | 0.008 | 0.144 | 0.000 | 0.575 | 0.011 | 0.174 | 0.001 | 0.088 | 0.001 | 0.669 | 0.014 |
| 5630bF | NN2110Bto3bF | 0.615 | 0.017 | 0.214 | 0.003 | 0.909 | 0.010 | 0.205 | 0.023 | 0.136 | 0.002 | 0.726 | 0.029 |
| 5630bH* | NN2110Bto3bH* | 0.557 | 0.010 | 0.151 | 0.000 | 0.633 | 0.004 | 0.175 | 0.003 | 0.101 | 0.004 | 0.552 | 0.019 |
| 5631bF | NN2110Bto4bF | 0.620 | 0.009 | 0.236 | 0.007 | 0.942 | 0.014 | 0.251 | 0.012 | 0.149 | 0.005 | 0.715 | 0.046 |
| 5631bH* | NN2110Bto4bH* | 0.526 | 0.005 | 0.152 | 0.000 | 0.574 | 0.018 | 0.185 | 0.007 | 0.101 | 0.001 | 0.603 | 0.020 |
| 5632bF | NN2230Bto1bF | 0.661 | 0.015 | 0.303 | 0.066 | 0.951 | 0.017 | 0.282 | 0.000 | 0.174 | 0.000 | 0.606 | 0.000 |
| 5632bH* | NN2230Bto1bH* | 0.562 | 0.006 | 0.184 | 0.004 | 0.630 | 0.005 | 0.205 | 0.003 | 0.111 | 0.002 | 0.538 | 0.025 |
| 5633bF | NN2285Bto1bF | 0.648 | 0.020 | 0.230 | 0.012 | 0.969 | 0.007 | 0.273 | 0.004 | 0.169 | 0.000 | 0.725 | 0.008 |
| 5633bH* | NN2285Bto1bH* | 0.534 | 0.005 | 0.164 | 0.002 | 0.589 | 0.002 | 0.193 | 0.002 | 0.096 | 0.001 | 0.582 | 0.006 |
| 5634bF | NN2285Bto2bF | 0.656 | 0.018 | 0.270 | 0.034 | 0.986 | 0.001 | 0.271 | 0.007 | 0.169 | 0.002 | 0.687 | 0.004 |
| 5634bH* | NN2285Bto2bH* | 0.522 | 0.008 | 0.158 | 0.001 | 0.577 | 0.003 | 0.185 | 0.001 | 0.097 | 0.001 | 0.567 | 0.000 |
| 5635bF | NN2285Bto3bF | 0.664 | 0.022 | 0.260 | 0.006 | 0.967 | 0.028 | 0.290 | 0.008 | 0.188 | 0.005 | 0.627 | 0.045 |
| 5635bH* | NN2285Bto3bH* | 0.546 | 0.008 | 0.169 | 0.003 | 0.594 | 0.010 | 0.201 | 0.001 | 0.106 | 0.002 | 0.526 | 0.017 |
| 5636bF | NN2285Bto4bF | 0.655 | 0.003 | 0.217 | 0.020 | 0.924 | 0.008 | 0.281 | 0.006 | 0.154 | 0.002 | 0.730 | 0.013 |
| 5636bH* | NN2285Bto4bH* | 0.535 | 0.004 | 0.164 | 0.001 | 0.567 | 0.008 | 0.215 | 0.002 | 0.097 | 0.001 | 0.511 | 0.018 |
| 5637bF | NN2315Bto1bF | 0.675 | 0.000 | 0.251 | 0.019 | 0.962 | 0.024 | 0.280 | 0.001 | 0.176 | 0.004 | 0.655 | 0.028 |
| 5637bH* | NN2315Bto1bH* | 0.573 | 0.010 | 0.176 | 0.000 | 0.649 | 0.014 | 0.215 | 0.004 | 0.112 | 0.000 | 0.504 | 0.012 |
| 5638bF | NN2315Bto2bF | 0.662 | 0.003 | 0.248 | 0.015 | 0.964 | 0.011 | 0.269 | 0.004 | 0.170 | 0.003 | 0.652 | 0.015 |
| 5638bH* | NN2315Bto2bH* | 0.540 | 0.008 | 0.169 | 0.002 | 0.641 | 0.006 | 0.193 | 0.001 | 0.106 | 0.002 | 0.532 | 0.040 |
| 5639bF | NN2315Bto3bF | 0.648 | 0.001 | 0.238 | 0.003 | 0.946 | 0.007 | 0.289 | 0.005 | 0.159 | 0.001 | 0.684 | 0.006 |
| 5639bH* | NN2315Bto3bH* | 0.521 | 0.000 | 0.161 | 0.000 | 0.564 | 0.001 | 0.218 | 0.005 | 0.104 | 0.002 | 0.593 | 0.001 |
| 5640bF | NN2395Bto1bF | 0.643 | 0.002 | 0.248 | 0.026 | 0.929 | 0.013 | 0.279 | 0.003 | 0.168 | 0.008 | 0.819 | 0.021 |
| 5640bH* | NN2395Bto1bH* | 0.529 | 0.006 | 0.161 | 0.000 | 0.599 | 0.004 | 0.195 | 0.001 | 0.097 | 0.002 | 0.635 | 0.008 |
| 5641bF | NN2395Bto2bF | 0.648 | 0.000 | 0.231 | 0.021 | 0.947 | 0.004 | 0.247 | 0.001 | 0.182 | 0.005 | 0.710 | 0.014 |
| 5641bH* | NN2395Bto2bH* | 0.497 | 0.010 | 0.155 | 0.002 | 0.565 | 0.006 | 0.170 | 0.001 | 0.090 | 0.005 | 0.579 | 0.025 |
| 5642bF | NN2395Bto1bF | 0.635 | 0.000 | 0.251 | 0.020 | 0.753 | 0.013 | 0.277 | 0.002 | 0.172 | 0.009 | 0.708 | 0.014 |
| 5642bH* | NN2395Bto1bH* | 0.482 | 0.002 | 0.158 | 0.004 | 0.474 | 0.001 | 0.191 | 0.002 | 0.097 | 0.001 | 0.519 | 0.050 |
| 5643bF | NN2445Bto1bF | 0.652 | 0.017 | 0.239 | 0.011 | 0.931 | 0.020 | 0.273 | 0.004 | 0.169 | 0.001 | 0.639 | 0.000 |
| 5643bH* | NN2445Bto1bH* | 0.578 | 0.001 | 0.199 | 0.000 | 0.847 | 0.021 | 0.248 | 0.025 | 0.160 | 0.001 | 0.730 | 0.037 |
| 5644bF | NN2445Bto2bF | 0.657 | 0.023 | 0.258 | 0.007 | 0.987 | 0.006 | 0.271 | 0.005 | 0.173 | 0.006 | 0.667 | 0.002 |
| 5644bH* | NN2445Bto2bH* | 0.525 | 0.008 | 0.167 | 0.007 | 0.596 | 0.008 | 0.202 | 0.019 | 0.104 | 0.004 | 0.527 | 0.016 |
| 5645bF | NN2445Bto3bF | 0.649 | 0.018 | 0.270 | 0.052 | 0.971 | 0.016 | 0.265 | 0.002 | 0.161 | 0.004 | 0.693 | 0.017 |
| 5645bH* | NN2445Bto3bH* | 0.500 | 0.004 | 0.159 | 0.006 | 0.555 | 0.006 | 0.198 | 0.021 | 0.098 | 0.006 | 0.492 | 0.053 |
| 5646bF | NN2445Bto4bF | 0.639 | 0.005 | 0.199 | 0.010 | 0.922 | 0.015 | 0.279 | 0.001 | 0.153 | 0.015 | 0.720 | 0.002 |
| 5646bH* | NN2445Bto4bH* | 0.528 | 0.005 | 0.164 | 0.002 | 0.598 | 0.017 | 0.217 | 0.008 | 0.109 | 0.001 | 0.580 | 0.024 |
| 5647bF | NN2505Bto1bF | 0.650 | 0.019 | 0.241 | 0.000 | 0.952 | 0.009 | 0.264 | 0.004 | 0.162 | 0.004 | 0.651 | 0.022 |
| 5647bH* | NN2505Bto1bH* | 0.531 | 0.005 | 0.164 | 0.005 | 0.592 | 0.006 | 0.201 | 0.016 | 0.104 | 0.003 | 0.510 | 0.007 |
| 5648bF | NN2505Bto2bF | 0.660 | 0.021 | 0.259 | 0.018 | 0.936 | 0.006 | 0.278 | 0.003 | 0.188 | 0.001 | 0.624 | 0.001 |
| 5648bH* | NN2505Bto2bH* | 0.550 | 0.006 | 0.166 | 0.000 | 0.629 | 0.005 | 0.216 | 0.014 | 0.123 | 0.005 | 0.460 | 0.037 |
| 5649bF | NN2505Bto3bF | 0.653 | 0.001 | 0.220 | 0.032 | 0.942 | 0.017 | 0.295 | 0.002 | 0.163 | 0.001 | 0.583 | 0.015 |
| 5649bH* | NN2505Bto3bH* | 0.534 | 0.007 | 0.160 | 0.004 | 0.592 | 0.008 | 0.209 | 0.015 | 0.108 | 0.004 | 0.476 | 0.011 |
| 5650bF | NN2565Bto1bF | 0.659 | 0.005 | 0.230 | 0.004 | 0.960 | 0.005 | 0.273 | 0.001 | 0.107 | 0.065 | 0.612 | 0.018 |
| 5650bH* | NN2565Bto1bH* | 0.534 | 0.002 | 0.172 | 0.001 | 0.615 | 0.005 | 0.211 | 0.000 | 0.115 | 0.001 | 0.513 | 0.008 |
| 5687bF | NN2565Bto2bF | 0.642 | 0.004 | 0.215 | 0.009 | 0.917 | 0.024 | 0.290 | 0.007 | 0.164 | 0.007 | 0.716 | 0.027 |
| 5687bH* | NN2565Bto2bH* | 0.486 | 0.002 | 0.146 | 0.001 | 0.526 | 0.001 | 0.191 | 0.010 | 0.101 | 0.006 | 0.617 | 0.006 |
| 5688bF | NN2565Bto3bF | 0.648 | 0.007 | 0.229 | 0.005 | 0.906 | 0.023 | 0.280 | 0.013 | 0.176 | 0.008 | 0.625 | 0.008 |
| 5688bH* | NN2565Bto3bH* | 0.504 | 0.004 | 0.156 | 0.001 | 0.523 | 0.005 | 0.203 | 0.004 | 0.106 | 0.006 | 0.519 | 0.006 |
| 5689bF | NN2565Bto4bF | 0.632 | 0.002 | 0.238 | 0.001 | 0.907 | 0.003 | 0.275 | 0.000 | 0.171 | 0.008 | 0.565 | 0.007 |
| 5689bH* | NN2565Bto4bH* | 0.517 | 0.001 | 0.159 | 0.001 | 0.565 | 0.010 | 0.206 | 0.007 | 0.101 | 0.004 | 0.471 | 0.012 |
| 5690bF | NN2590Bto1bF | 0.635 | 0.009 | 0.218 | 0.023 | 0.875 | 0.027 | 0.281 | 0.013 | 0.173 | 0.001 | 0.773 | 0.005 |
| 5690bH* | NN2590Bto1bH* | 0.485 | 0.005 | 0.150 | 0.003 | 0.513 | 0.010 | 0.200 | 0.003 | 0.101 | 0.003 | 0.566 | 0.104 |
| 5691bF | NN2590Bto2bF | 0.644 | 0.004 | 0.207 | 0.024 | 0.946 | 0.007 | 0.267 | 0.005 | 0.155 | 0.008 | 0.704 | 0.016 |
| 5691bH* | NN2590Bto2bH* | 0.489 | 0.002 | 0.144 | 0.008 | 0.515 | 0.019 | 0.195 | 0.001 | 0.107 | 0.003 | 0.565 | 0.008 |
| 5692bF | NN2590Bto3bF | 0.643 | 0.006 | 0.237 | 0.017 | 0.940 | 0.050 | 0.282 | 0.001 | 0.177 | 0.012 | 0.652 | 0.000 |
| 5692bH* | NN2590Bto3bH* | 0.491 | 0.004 | 0.146 | 0.001 | 0.539 | 0.008 | 0.197 | 0.008 | 0.102 | 0.001 | 0.492 | 0.084 |
| 5693bF | NN2590Bto4bF | 0.631 | 0.005 | 0.222 | 0.019 | 0.904 | 0.020 | 0.287 | 0.001 | 0.171 | 0.008 | 0.747 | 0.013 |
| 5693bH* | NN2590Bto4bH* | 0.480 | 0.002 | 0.136 | 0.005 | 0.532 | 0.005 | 0.186 | 0.003 | 0.098 | 0.007 | 0.651 | 0.008 |
| 5694bF | NN2A1Bto1bF | 0.642 | 0.005 | 0.216 | 0.005 | 0.923 | 0.013 | 0.253 | 0.007 | 0.158 | 0.001 | 0.720 | 0.008 |
| 5694bH* | NN2A1Bto1bH* | 0.511 | 0.004 | 0.156 | 0.002 | 0.566 | 0.005 | 0.210 | 0.001 | 0.057 | 0.072 | 0.555 | 0.015 |
| 5695bF | NN2A1Bto2bF | 0.637 | 0.004 | 0.189 | 0.007 | 0.914 | 0.014 | 0.241 | 0.012 | 0.148 | 0.006 | 0.678 | 0.014 |
| 5695bH* | NN2A1Bto2bH* | 0.522 | 0.002 | 0.155 | 0.000 | 0.596 | 0.004 | 0.193 | 0.007 | 0.056 | 0.072 | 0.607 | 0.002 |
| 5696bF | NN2A1Bto3bF | 0.641 | 0.011 | 0.217 | 0.024 | 0.912 | 0.027 | 0.275 | 0.006 | 0.157 | 0.003 | 0.699 | 0.021 |
| 5696bH* | NN2A1Bto3bH* | 0.530 | 0.004 | 0.160 | 0.001 | 0.579 | 0.013 | 0.203 | 0.004 | 0.108 | 0.013 | 0.531 | 0.009 |
| 5697bF | NN2A1Bto4bF | 0.638 | 0.004 | 0.215 | 0.017 | 0.931 | 0.009 | 0.249 | 0.003 | 0.151 | 0.008 | 0.706 | 0.025 |
| 5697bH* | NN2A1Bto4bH* | 0.507 | 0.002 | 0.152 | 0.003 | 0.567 | 0.010 | 0.197 | 0.012 | 0.096 | 0.002 | 0.584 | 0.009 |

**Thermoluminescence dating (D. Richter)**

Thermoluminescence (TL) dating of heated flint artefacts is a well established method for dating Middle Palaeolithic archaeological sites (Valladas et al., 1988). A past human activity can be directly dated by establishing the time elapsed since a flint artefact fell into a prehistoric fire. TL dating is based on the accumulation of electrons in excited states in the crystal lattice due to omnipresent ionizing radiation. These metastable states can be quantitatively measured by TL analysis and provide the dose the sample has received since its last zeroing (heating) in the fire when all excited states were eliminated by the prehistoric heating. Standard analysis of TL dating of heated flints is based on multiple aliquot techniques with a combined additive and regeneration (MAAR) protocol (Mercier et al., 1991; Valladas et al., 1988).

This standard approach requires sample weights of several grams, which are not available at Neumark-Nord 2. Almost all flints that were macroscopically identified as having been burnt (craquelation, potlids, ect.) were in the weight range of 2-6 g and thus too small for standard TL-dating approaches. Large samples are required because for dosimetric reasons the outer two mm of samples are removed with a water-cooled diamond saw, resulting in the loss of a large amount of material of such irregularly shaped objects like flint artefacts (Richter, 2007). Therefore a single aliquot regeneration (SAR) technique had to be used, which requires in principle only a few mg of sample material after extraction (removal). This new approach employed here makes use of the almost absent sensitivity change of the TL signal of quartz in the orange-red wavelength detection band, which is also observed for many flints that basically consist of cryptocrystalline quartz. Because of these negligible sensitivity changes no test dose measurements are required (Richter and Krbetschek, 2006). The standard SAR technique can be even further reduced to the measurement of two regeneration points only (Richter and Krbetschek, 2006), which greatly reduces the time needed for measuring large palaeodoses in the laboratory. However, as a check of the assumption of negligible sensitivity change a repetition of one regeneration dose point was measured as well. The palaeodose is therefore derived from the level of the natural TL signal as defined by the intersection of this level with a straight line connecting the measurement results (TL) of two regeneration doses. If the integrated TL (as defined by the temperature range of the heating plateau) from these two regeneration dose points are close to the natural TL, any deviation of the regeneration dose response curve from a straight line is negligible (Richter, 2009). In any case the doses for samples at Neumark-Nord 2 are still well in the linear dose range of flint and therefore this issue is of no concern here and the dose response curves can be assumed as being linear in the range under investigation here. The validity of this short SAR approach was shown for different samples in the linear as well as sublinear dose range for several sites and materials (Richter and Temming, 2006).

For each sample between 12 and 24 aliquots of 4 mg were measured in order to obtain statistically more valid results. Rejection criteria for individual aliquots were defined as recycling ratios between 0.85-1.15, a palaeodose error of less than 10% and the natural TL. had to be bracketed by the two regeneration dose points. An additional rejection criterion was defined as a maximum deviation of 20% of the regenerated TL from the natural TL. Failure of this criterion is a reflection of the poor knowledge of the approximate natural dose and the subsequent misjudgement of the doses to be applied by the operator. However, as these samples are certainly still well within the linear range of their TL growth curves a regeneration response beyond 20% appears to be acceptable, but there are no means to objectively determine a threshold. Subsequent analysis with a higher threshold increased the number of acceptable aliquots significantly and produced virtually identical results. The samples were measured in groups of 5-10 aliquots and knowledge of regeneration doses increased with aliquot number and led to a reduction of rejected aliquots with time. Therefore large numbers of rejected aliquots on basis of failure of this criterion are not a reflection of ‘bad’ behaviour of the samples but rather of the lack of experience with this new protocol.

Because flints contain natural radioactive elements their, incontestably stable, contribution to the total dose rate needs to be taken into account. Samples for Neutron Activation Analysis (NAA) to determine U, Th and K were taken from crushed material of the extracted core of the samples prior to chemical treatment. This internal dose rate is established by the determination of the alpha sensitivity (b-value) for each sample separately by comparing the TL yield from calibrated alpha and beta sources on fine grained (4-11 µm) material which was zeroed for 30 min at 500°C.

The external γ-dose rate was determined *in situ* in the excavation sections over a period of several months by five Al_2_O_3_:C dosemeters because of the heterogeneity of the sediments containing the archaeological material and the varying thickness of the archaeological deposit. The results from these dosemeters display a variability of 10% standard deviation from the mean and therefore such an uncertainty for the external γ-dose rate was employed for the dosemeter average of 956 µGy a^-1^, which is confirmed by moisture corrected HpGe-γ-ray-spectrometry. The sediments from this deposit as well as those above and below were measured by HpGe-γ-ray spectrometry in order to determine that the U- and Th-series are in secular equilibrium and therefore the assumption of stability of the external γ-dose rate over time is valid. The external γ-dose rate was adjusted for sample size, but no adjustments were made for water content. A cosmic dose of 90 µGy a^-1^ was estimated according to the geographic position and sediment thickness, resulting in an effective external γ-dose rate of 1046 µGy a^-1^ for the samples.

Table S3: Results of NAA and TL measurements.

| Sample | U | Th | K | b-value | effective internal dose rate | total dose rate | Number of accepted aliquots | average recycling ratio | palaeodose (Gy) | age (ka) |
| --- | --- | --- | --- | --- | --- | --- | --- | --- | --- | --- |
| NN2-001 | 0.23±0.04 | 0.13±0.02 | 510±20 | 0.697 | 80±6 | 1117±95 | 23 | 1.13±0.02 | 140±6 | 125±12 |
| NN2-002 | 0.35±0.04 | 0.09±0.01 | 290±20 | 0.731 | 81±6 | 1117±96 | 14 | 1.03±0.02 | 150±6 | 134±13 |
| NN2-005 | 0.40±0.04 | 0.11±0.02 | 445±27 | 0.611 | 101±7 | 1137±95 | 15 | 1.03±0.01 | 153±4 | 135±13 |
| NN2-009 | 0.50±0.05 | 0.12±0.02 | 308±22 | 0.805 | 108±8 | 1144±95 | 17 | 1.03±0.01 | 140±3 | 122±12 |
| NN2-018 | 0.31±0.03 | 0.12±0.02 | 356±25 | 0.826 | 81±6 | 1118±95 | 17 | 1.06±0.03 | 133±7 | 119±12 |

The resulting palaeodoses display a smaller variation than the observed external γ-dose rate, which is an indirect indication of the validity of the latter parameter. The age results cluster narrowly between 119 and 135 ka and belong to a normal distribution. This allows the calculation of a weighted average age of 126 ± 6 ka, which best represents the time of the last heating of these flint artefacts and testifies the time of the activity of lighting a fire by Neandertals during the Eemian at Neumark-Nord 2.

**Paleomagnetic procedures (M.J. Sier & M.J. Dekkers)**

Sampling

Dedicated perspex sample containers with standard dimension (25 mm diameter and 22 mm height) were gently pushed into freshly prepared cut-outs in the section. After orientation with a magnetic compass and inclinometer they were taken out from the section and closed with a plastic lid and sealed for measurement in paleomagnetic laboratory ‘Fort Hoofddijk’ (Utrecht University, Utrecht, The Netherlands). Measurements were done within one month after retrieval of the samples to ensure that the samples were processed while still fresh. For thermal demagnetization an electrical drill powered by a portable generator was used to drill the fragile sediments on selected intervals. Orientation was done with a magnetic compass and a dedicated orientation device. Only the most sandy layers could not be drilled, cores desintegrated due to the water used to flush away the drilled sediment. All samples were processed within 14 days after collection. For the demagnetization standard-sized specimens were cut with a plastic knife from the drill cores; subsequently they were wrapped in aluminium foil to withstand the manipulating that jeopardizes the physical stability of the samples during thermal treatment.

Demagnetization

Alternating field (AF) demagnetization in 15 steps to up 100 mT was carried out with a robotized direct current superconducting quantum interference device (DC-SQUID) magnetometer manufactured by the ‘2G’ company (Mountain View California, USA). The instrument sensitivity is 3x10^-12^ Am^2^ and typical sample intensities were at least two orders of magnitude higher. Static three-axial AF demagnetization was done with a so-called in-line AF demagnetization coil set directly attached to the magnetometer. The instrument set-up is housed inside a magnetically shielded room (residual field < 200 nT); the robotized interface for sample manipulation was built in-house. Up to 96 samples contained in dedicated cubic holders (edge 30 mm) are loaded onto a sample plateau and the robot loads them in batches of eight onto a tray that can be slided through the magnetometer and demagnetization coils. Samples are processed fully automatically with the so-called ‘three position protocol’ that compensates for the magnetic moment of the transport tray. This ensures optimal processing of weakly magnetic samples.

Thermal demagnetization was performed with in-house constructed thermal demagnetizers (residual field < 50 nT) and measured with another DC-SQUID magnetometer (instrument sensitivity 5x10^-12^ Am^2^). During thermal demagnetization the samples appeared to become highly viscous after demagnetization at temperatures > ~350°C due to neoformation of extremely fine-grained magnetite (grain size ~20 nm). To have the viscous moment decayed entirely a waiting time of 5 to 10 minutes was implemented with the sample inside the magnetometer in a very low ambient field before each instrument reading was taken. Only by processing with such a long waiting meaningful demagnetization results could be obtained. To avoid the development of disturbing viscous magnetic moments during the thermal demagnetization a sample set was thermally demagnetized up to 205°C to demagnetize the natural viscous component acquired in ~100 ka in the geomagnetic field. After this thermal treatment they were further demagnetized by AF demagnetization in the robotized magnetometer system.

Demagnetization data are plotted in orthonormal vector endpoint diagrams or Zijderveld diagrams (Zijderveld, 1967). Representative examples are shown in figure S7. Sample Neu117 is processed by AF demagnetization, it shows a large overprint and a small characteristic remanent magnetization (ChRM) with a transitional/reversed direction. The large overprint is caused by viscous overprinting but also by physical realignment of the particles in the strong geomagnetic field that prevailed directly after the Blake Event (cf. Coe and Liddicoat’s (Coe and Liddicoat, 1994) explanation for features of the Mono Lake excursion record). The Blake Event is like all geomagnetic events characterized by a weak geomagnetic field intensity. Sample Neu219 that is treated with AF demagnetization shows the development of so-called gyroremanent magnetization (GRM) (Stephenson, 1993), a characteristic property of greigite (Fe_3_S_4_) a magnetic mineral that may often occur in fresh-water sediments (e.g. (Hu et al., 2002; Snowball, 1997)). The greigite is formed in-situ over an undefined duration albeit that most greigite NRM is acquired shortly after deposition (i.e. within ~10 ka). The potential presence of greigite thus provides an other possibility for having large normal overprints potentially obscuring detection of the detrital ChRM component. Indeed during thermal demagnetization of a sister specimen most NRM is demagnetized at ~350°C, at the end of the temperature interval during which greigite chemically alters to a non-magnetic ferrous-ferric sulfate (~220-~350°C, e.g.(Dekkers et al., 2000)). Also Neu 237 with its easterly declination shows this behaviour, its direction could be a composite of a transitional direction with a later normal overprint caused by prolonged greigite formation. Samples Neu 253 and Neu 255 are AF demagnetized after thermal demagnetization to 205°C and indeed the determination of the ChRM is more straightforward than with AF demagnetization only. Of course for levels devoid of greigite observed NRM directions may be representative of the (non-dipole) transitional geomagnetic field during an excursion. However, overprinting of a strong normal direction on the weak transitional ChRM may occur as well.

To discriminate between directional scatter due to secular variation of the geomagnetic field and excursional directions, the data set was subjected to the variable cut-off procedure (Vandamme, 1994). Samples with deviating directions appeared to be stratigraphically coherent and are interpreted to derive from the Blake Event.

Delayed NRM acquisition?

The paleomagnetic age assignment of the Blake Event hinges on the quality of its recording in the sediments which is generally not straightforward. Post-depositional processes may obscure the recording and its preservation. This includes pDRM lock-in aspects that can be lithology-dependent (Hofmann and Fabian, 2007; Tauxe, 1993) post-depositional realignment in stronger post-Blake geomagnetic fields (Coe and Liddicoat, 1994), biological activity (Egli, 2004; Kruiver and Passier, 2001) and a myriad of early diagenetic processes (e.g. Calvert and Fontugne, 2001; Passier et al., 2001; Rowan and Roberts, 2006). Recording of geomagnetic events in high-latitude Arctic cores has recently been shown suspect in cases where the events are associated to horizons containing greigite that would have acquired a self-reversed NRM (Channell and Xuan, 2009). Also the loess archive on the Chinese Loess Plateau should be scrutinized before a recording of a geomagnetic excursion can be assessed as genuine: for the Blake Event Zhu et al. (Zhu et al., 1998) document a limited recording or even non-recording (or preservation) as a function of interfering pedogenesis or discontinuous loess deposition due to erosive storm events. Nonetheless there seem to exist recordings of the Blake Event that are less complicated in this sense in open ocean settings with a limited geochemical contrast in ODP Site 1062 on the Blake/Bahama Outer Ridge (Lund et al., 2006). These authors assign an age slightly older than 120 ka to the event but a record of the transition itself was not provided. The Blake Event in the Gardar Drift sediments (Channell et al., 1997) at ~119 ka has a short-lived declination swing but its inclination record appears to be subdued. On the basis of their study of a set of cores from the Kolbeinski Ridge (Iceland Sea), Nowaczyk and Frederichs (Nowaczyk and Frederichs, 1999) argue that the Blake Event is recorded as a double event straddling 120 ka. Zhu et al. (Zhu et al., 2000) determined K/Ar ages of 123 ± 7 and 123 ± 10 ka for two lavas with intermediate palaeomagnetic directions in the Tianchi volcanic sequence (NE China) that were associated to the Blake Event.

We base our terrestrial-marine correlation primarily on the Blake Event, notably that one as described in Tric et al. (Tric et al., 1991), because that is so detailed. Regarding the issue of a possible delayed NRM acquisition two aspects needs to be discussed: 1) The Tric et al. (Tric et al., 1991) record is from an interval more or less directly above a sapropel that may have been burnt down to some degree (Higgs et al., 1994; vanSantvoort et al., 1997). Delayed NRM acquisition would imply that the Blake Event would be younger than the sediment level of its recording, thus enhancing the discrepancy between the terrestrial expression of the Eemian and MIS 5e. 2) In the Neumark record delayed NRM acquisition could have occurred during a younger phase of the terrestrial Eemian.

1) Burnt-down sapropel levels often contain traces of magnetotactic magnetite (Kruiver and F. Passier, 2001), an aspect that could not be known by Tric et al. (1991) because it was not yet discovered. Therefore detailed IRM acquisition curves to track this possibility are not available for the Tric et al. (Tric et al., 1991) record. However, there is a detailed geochemical study available (Calvert and Fontugne, 2001) for a nearby core collected during the same cruise, core MD84641. Both cores are from the northernmost outer parts of the river Nile cone. The Tric et al. (Tric et al., 1991) record derives from core MD84627, but Tucholka et al. (Tucholka, 1987) found the Blake Event also in MD84641 albeit less well expressed (the reason that Tric et al. (Tric et al., 1991) focussed on core MD84627). Geochemical proxies (all from Calvert and Fontugne (Calvert and Fontugne, 2001)) from directly above sapropel S5, notably the Si/Al and Ti/Al ratios that are not influenced by diagenetic iron mobility, do not hint at an originally much thicker sapropel. Also sulfidic sulfur (in the form of pyrite grains precipitated) did not migrate downward from S4 all the way down to S5 as a possible consequence of later re-establishment of anoxic conditions during formation of sapropel S4. Also Ba/Al levels are not enriched above S5 in that core (note below S5 they seem to be ‘highish’). So, although some delayed NRM acquisition as a consequence of early diagenesis cannot be excluded, distinctly later diagenetic overprinting (with a delay of several thousands of years) is unlikely in the Tric et al. (Tric et al., 1991) record despite its close association to a sapropel. Also it is rather difficult to surmize how to generate a double geomagnetic event with the reported relatively smooth VGP path as a result of diagenetic imprint of a weak reversed geomagnetic field onto a normal polarity NRM that represents a stronger geomagnetic field (because at that time the field is not in a transitional state).

2) The possibility of delayed NRM acquisition in the Neumark record.

Also in the Neumark record delayed NRM acquisition could have occurred, particularly in darker coloured greigite-bearing strata where some delay in NRM acquisition coupled to a prolonged NRM acquisition duration is bound to have occurred. Sedimentation rates in the section are appreciable; therefore the time-span available for biologic and (very) early diagenetic processes is probably limited (most activity takes place close to the sediment-water interface). The paleomagnetic directional record does not correlate with (subtle) changes in lithology which makes diagenetic imprint of the entire Blake Event over a thickness of ~6 metres unlikely. Moreover, the artifact find-layer in the very same section has been dated to 126 ± 6 ka which concurs with an undelayed recording of the Blake Event in the Neumark succession.

**References Supplementary Material**

Bada, J.L., Shou, M.Y., Man, E.H. and Schroeder, R.A., 1978, Decomposition of hydroxy amino acids in foraminiferal tests: Kinetics, mechanism and geochronological implications. Earth and Planetary Science Letters, 41: 67-76.

Bowen, D.Q., 1999, A Revised Correlation of Quaternary Deposits in the British Isles. Geological Society of London, London

Calvert, S.E. and Fontugne, M.R., 2001, On the late Pleistocene-Holocene sapropel record of climatic and oceanographic variability in the eastern Mediterranean. Paleoceanography, 16: 78-94.

Channell, J.E.T., Hodell, D.A. and Lehman, B., 1997, Relative geomagnetic paleointensity and [delta]18O at ODP Site 983 (Gardar Drift, North Atlantic) since 350 ka. Earth and Planetary Science Letters, 153: 103-118.

Channell, J.E.T. and Xuan, C., 2009, Self-reversal and apparent magnetic excursions in Arctic sediments. Earth and Planetary Science Letters, In Press, Corrected Proof.

Coe, R.S. and Liddicoat, J.C., 1994, Overprinting of natural magnetic remanence in lake sediments by a subsequent high-intensity field. Nature, 367.

Dekkers, M.J., Passier, H.F. and Schoonen, M.A.A., 2000, Magnetic properties of hydrothermally synthesized greigite (Fe3S4) High- and low-temperature characteristics. Geophysical Journal International, 141: 809.

Egli, R., 2004, Characterization of individual rock magnetic components by analysis of remanence curves. 3. Bacterial magnetite and natural processes in lakes. Physics and Chemistry of the Earth, Parts A/B/C, 29: 869-884.

Eissmann, L., 2002, Quaternary geology of eastern Germany (Saxony, Saxon-Anhalt, South Brandenburg, Thüringia), type area of the Elsterian and Saalian Stages in Europe. Quaternary Science Reviews, 21: 1275-1346.

FAO-Unesco ed. 1988, World Soil Resources Report. Food and Agriculture Organization of the United Nations, Rome 138 p.

FAO ed. 1977, Guidelines for soil profile description. Food and Agriculture Organization of the United Nations, Rome 66 p.

Higgs, N.C., Thomson, J., Wilson, T.R.S. and Croudace, I.W., 1994, Modification and complete removal of eastern Mediterranean sapropels by postdepositional oxidation. Geology, 22: 423-426.

Hill, R.L., 1965, Hydrolysis of proteins. Advances in Protein Chemistry, 20: 37-107.

Hofmann, D.I. and Fabian, K., 2007, Rock magnetic properties and relative paleointensity stack for the last 300 ka based on a stratigraphic network from the subtropical and subantarctic South Atlantic. Earth and Planetary Science Letters, 260: 297-312.

Hu, S., Stephenson, A. and Appel, E., 2002, A study of gyroremanent magnetisation (GRM) and rotational remanent magnetisation (RRM) carried by greigite from lake sediments. Geophysical Journal International, 151: 469.

Kaufman, D.S. and Manley, W.F., 1998, A new procedure for determining DL amino acid ratios in fossils using Reverse Phase Liquid Chromatography. Quaternary Science Reviews, 17: 987-1000.

Kruiver, P.P. and F. Passier, H., 2001, Coercivity analysis of magnetic phases in sapropel S1 related to variations in redox conditions, including an investigation of the S ratio. Geochemistry Geophysics Geosystems, 2: 1063.

Laurat, T. and Brühl, E., 2006, Zum Stand der archäologischen Untersuchungen im Tagebau Neumark-Nord, Ldkr. Merseburg-Querfurt (Sachsen-Anhalt) - Vorbericht zu den Ausgrabungen 2003-2005. Jahresschrift für Mitteldeutsche Vorgeschichte, 90: 9-69.

Lund, S., Stoner, J.S., Channell, J.E.T. and Acton, G., 2006, A summary of Brunhes paleomagnetic field variability recorded in Ocean Drilling Program cores. Physics of the Earth and Planetary Interiors, 156: 194-204.

Mania, D., Thomae, M., Litt, T. and Weber, T., 1990, Neumark - Gröbern: Beiträge zur Jagd des mittelpaläolithischen Menschen. Veröffentlichungen des Landesmuseum für Vorgeschichte in Halle, 43: 1-319.

Mania, D. and Mania, U., 2008, Stratigraphy and Paleolithic of the Saale complex in the Elbe-Saale region. L'Anthropologie, 112: 15-47.

Menke, B. and Tynni, R., 1984, Das Eeminterglazial und das Weichselfrühglazial von Rederstall/Dittmarschen und ihre Bedeutung für die mitteleuropäische Jungpleistozängliederung. Geologisches Jahrbuch, A76: 3-120.

Mercier, N., Valladas, H., Joron, J.-L., Reyss, J.-L., Lévêque, F. and Vandermeersch, B., 1991, Thermoluminescence dating of the late Neanderthal remains from Saint-Césaire. Nature, 351: 737-739.

Nowaczyk, N.R. and Frederichs, T.W., 1999, Geomagnetic events and relative palaeointensity variations during the past 300ka as recorded in Kolbeinsey Ridge sediments, Iceland Sea: indication for a strongly variable geomagnetic field. International Journal of Earth Sciences, 88: 116-131.

Parfitt, S.A., Barendregt, R.W., Breda, M., Candy, I., Collins, M.J., Coope, G.R., Durbidge, P., Field, M.H., Lee, J.R., Lister, A.M., Mutch, R., Penkman, K.E.H., Preece, R.C., Rose, J., Stringer, C.B., Symmons, R., Whittaker, J.E., Wymer, J.J. and Stuart, A.J., 2005, The earliest record of human activity in northern Europe. Nature, 438: 1008-1012.

Passier, H.F., Lange, G.J.d. and Dekkers, M.J., 2001, Magnetic properties and geochemistry of the active oxidation front and the youngest sapropel in the eastern Mediterranean Sea. Geophysical Journal International, 145: 604-614.

Penkman, K.E.H., 2005, Amino Acid Geochronology: A Closed System Approach to Test and Refine the UK Model. Unpublished Ph.D. Thesis, University of Newcastle, UK

Penkman, K.E.H., Preece, R.C., Keen, D.H., Maddy, D., Schreve, D.C. and Collins, M.J., 2007, Testing the aminostratigraphy of fluvial archives: the evidence from intra-crystalline proteins within freshwater shells. Quaternary Science Reviews, 26: 2958-2969.

Penkman, K.E.H., Kaufman, D.S., Maddy, D. and Collins, M.J., 2008a, Closed-system behaviour of the intra-crystalline fraction of amino acids in mollusc shells. Quaternary Geochronology, 3: 2-25.

Penkman, K.E.H., Preece, R.C., Keen, D.H. and Collins, M.J., 2008b, British aggregates - An improved chronology using amino acid racemization and degradation of intra-crystalline amino acids (IcPD). English Heritage Research Department Report Series.

Penkman, K.E.H., Preece, R.C., Keen, D.H. and Collins, M.J., 2009, Amino acid geochronology of the type Cromerian of West Runton, Norfolk, UK. Quaternary International, in press.

Preece, R.C. and Penkman, K.E.H., 2005, New faunal analyses and amino acid dating of the Lower Palaeolithic site at East Farm, Barnham, Suffolk. Proceedings of the Geologists' Association, 116: 363-377.

Richter, D. and Krbetschek, M., 2006, A new Thermoluminescence dating technique for heated flint. Archaeometry, 48: 695-705.

Richter, D. and Temming, H., 2006, Testing heated flint palaeodose protocols using dose recovery procedures. Radiation Measurements, 41: 819-825.

Richter, D., 2007, Advantages and limitations of thermoluminescence dating of heated flint from Paleolithic sites. Geoarchaeology 22: 671-683.

Richter, D., 2009, Burnt flint artifacts: A new Thermoluminescence dating technique. Preistoria Alpina, 44: 41-55.

Rowan, C.J. and Roberts, A.P., 2006, Magnetite dissolution, diachronous greigite formation, and secondary magnetizations from pyrite oxidation: Unravelling complex magnetizations in Neogene marine sediments from New Zealand. Earth and Planetary Science Letters, 241: 119-137.

Snowball, I.F., 1997, Gyroremanent magnetization and the magnetic properties of greigite-bearing clays in southern Sweden. Geophysical Journal International, 129.

Stephenson, A., 1993, Three-Axis static alternating field demagnetisation of rocks and the identification of natural remanent magnetisation, gyroremanent magnetisation, and anisotropy. Journal of Geophysical Research, 98: 373.

Stockmarr, J., 1971, Tablets with spores used in absolute pollen analysis. Pollen Spores, 13: 615-621.

Stoops, G., 2003, Guidelines for Analysis and Description of Soil and Regolith Thin section. Soil Science Society of America, Madison

Sykes, G.A., Collins, M.J. and Walton, D.I., 1995, The significance of a geochemicaly isolated intracrystalline organic fraction within biominerals. Organic Geochemistry, 23: 1039-1065.

Tauber, H., 1965, Differential pollen dispersal and the interpretation of pollen diagrams. Danm. Geol. Unders. , II R: 89.

Tauber, H., 1967, Investigations of the mode of pollen transfer in forested areas. Rev. Palaeobot. Palynol., 3: 277-286.

Tauxe, L., 1993, Sedimentary records of relative paleointensity of the geomagnetic field - theory and practice. Reviews of Geophysics, 31: 319-354.

Thomae, M., 2003, Mollisoldiapirismus – Ursache für die Erhaltung der Fundstätte Neumark-Nord (Geiseltal). In: Burdukiewicz, J.M., Fiedler, L., Heinrich, W.D., Justus, A. and Brühl, E. (eds.), Erkenntnisjäger. Kultur und Umwelt des frühen Menschen. Landesambt für Archäologie Sachsen-Anhalt, Halle, 601-605.

Tric, E., Laj, C., Valet, J., Tucholka, P., Paterne, M. and Guichard, F., 1991, The Blake geomagnetic event: transition geometry, dynamical characteristics and geomagnetic significance. Earth and Planetary Science Letters, 102: 1-13.

Tucholka, P., Fontugne, M., Guichard, F., Paterne, M., 1987, The Blake magnetic polarity episode in cores from the Mediterranean Sea. Earth and Planetary Science Letters, 86: 320-326.

Turner, C. and West, R.G., 1968, The subdivision and zonation of interglacial periods. Eiszeitalter und Gegenwart, 19.

Turner, C., 2000, The Eemian interglacial in the North European plain and adjacent areas. Geologie en Mijnbouw - Netherlands Journal of Geosciences, 79 (2/3): 217-231.

Turner, C., 2002, Formal Status and Vegetational development of the Eemian Interglacial in Northwestern and Southern Europe. Quaternary Research, 58: 41-44.

Valladas, H., Reyss, J.L., Joron, J.L., Valladas, G., Bar-Yosef, O. and Vandermeersch, B., 1988, Thermoluminescence dating of mousterian 'Proto-Cro-Magnon' remains from Israel and the origin of modern man. Nature, 331: 614-616.

Vandamme, D., 1994, A new method to determine paleosecular variation. Physics of the Earth and Planetary Interiors, 85: 131.

vanSantvoort, P.J.M., deLange, G.J., Langereis, C.G., Dekkers, M.J. and Paterne, M., 1997, Geochemical and paleomagnetic evidence for the occurrence of ''missing'' sapropels in eastern Mediterranean sediments. Paleoceanography, 12: 773-786.

Zagwijn, W.H., 1961, Vegetation, climate and radiocarbon datings in the late Pleistocene of the Netherlands: Part I. Eemian and Early Weichselian. Mededelingen van de Geologische Stichting. Nieuwe Serie, 14: 15-45.

Zhu, R., Coe, R.S., Guo, B., Anderson, R. and Zhao, X., 1998, Inconsistent palaeomagnetic recording of the Blake event in Chinese loess related to sedimentary environment. Geophysical Journal International, 134: 867-875.

Zhu, R.X., Pan, Y.X. and Coe, R.S., 2000, Paleointensity studies of a lava succession from Jilin Province, northeastern China: Evidence for the Blake event. Journal of Geophysical Research-Solid Earth, 105: 8305-8317.

Zijderveld, J.D.A., 1967, Demagnetisation of rocks: analysis of results. In: Collinson, D.W., Creer, K.M. and Runcorn, S.K. (eds.), Methods in Palaeomagnetism. Elsevier, Amsterdam, 254-286.

**Figure S1**: overview of the NN 2/2 excavation, August 2008. A view from the north, on a E-W section perpendicular to the schematic section of Fig. 2. Left of the centre of the picture is an elephant tusk, in the middle of the archaeological find level NN2/2.


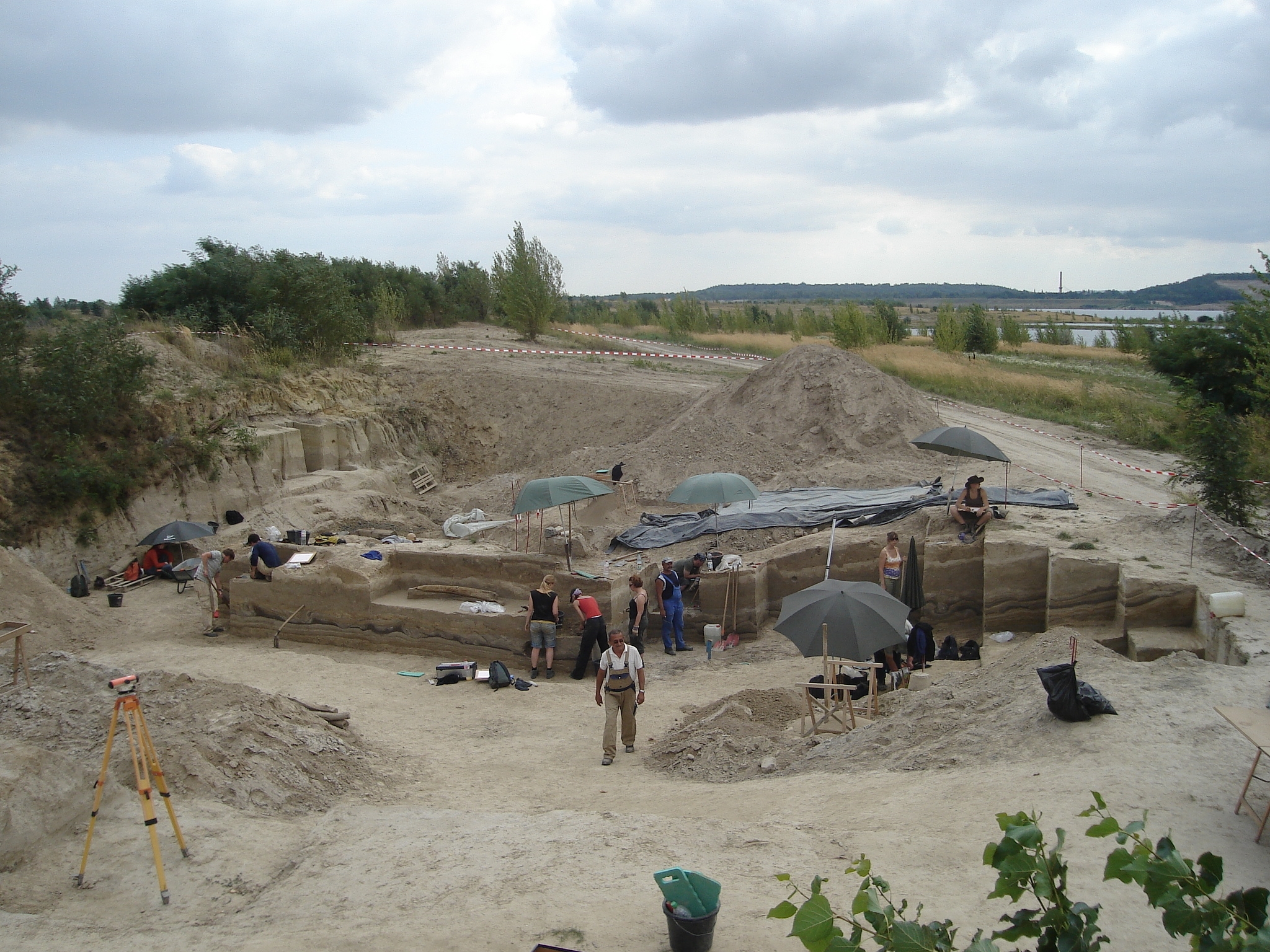


**Figure S2**: laminated sediment with crust formation and a micro fault (thin section M3lo-3, PPL).


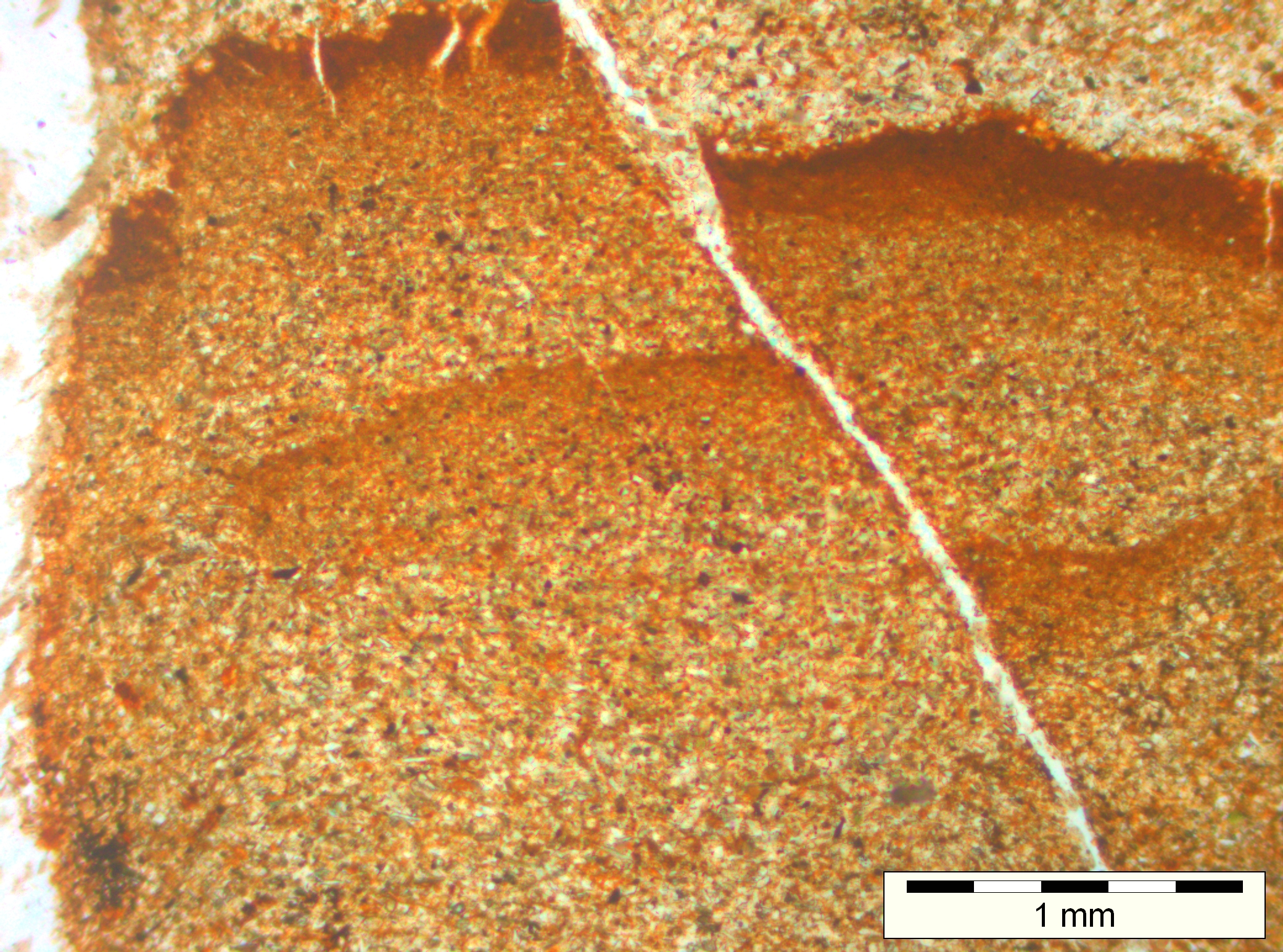


**Figure S3**: the same part of the thin section as Figure S3 in XPL.


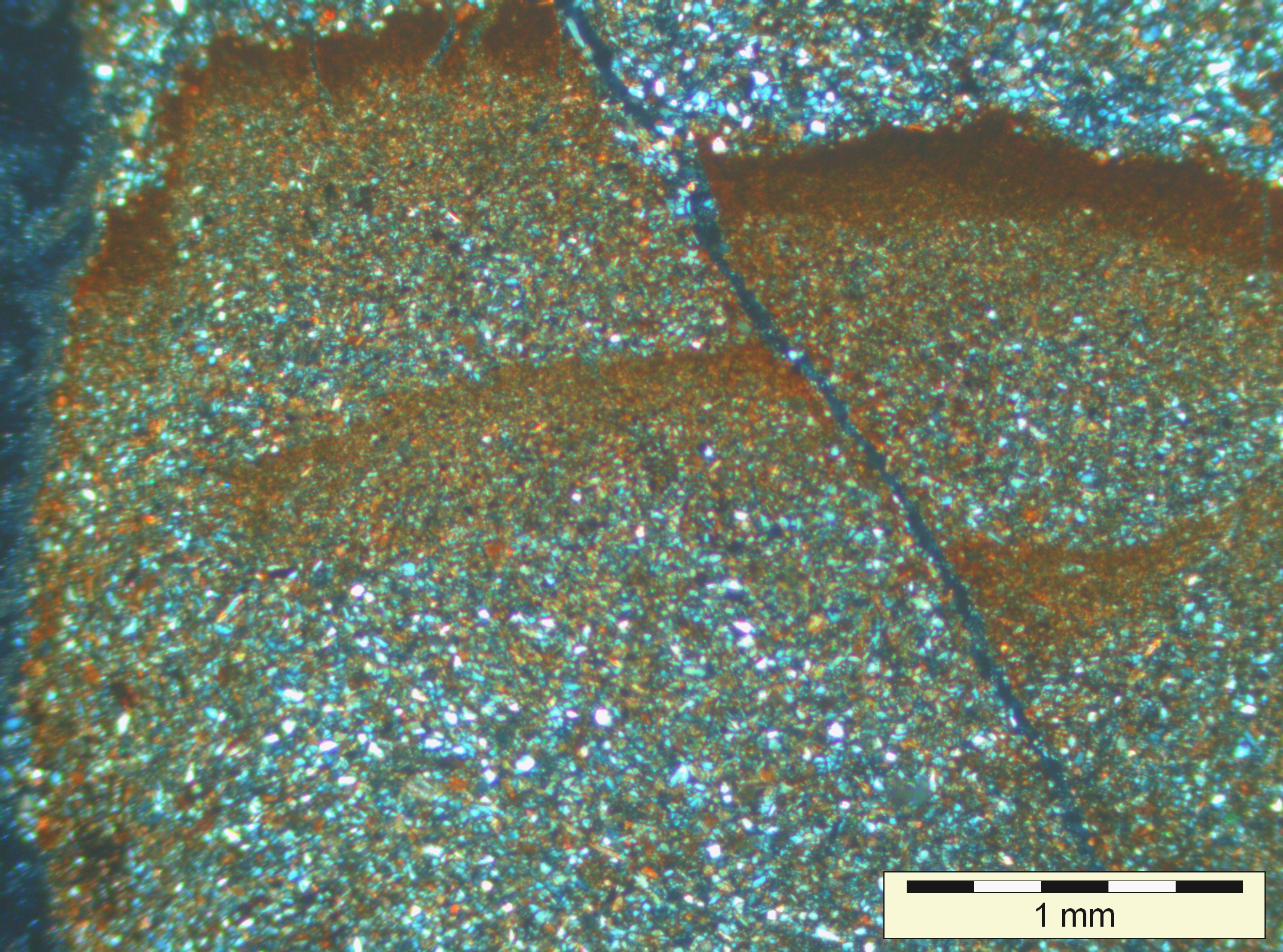


**Figure S4**: micro-rill with a thin clay layer at the bottom of the rill (thin section M3u-1, in PPL).


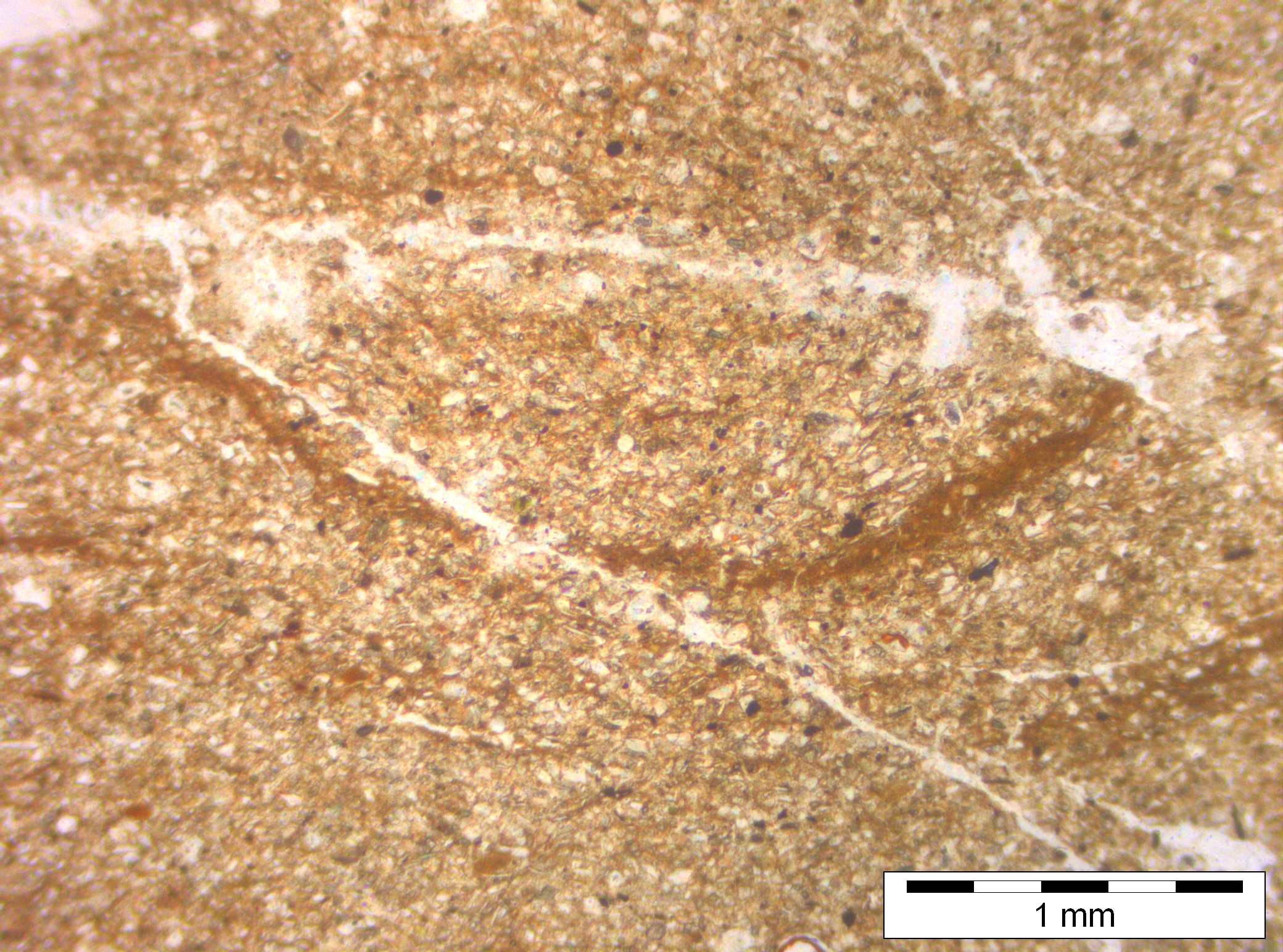


**Figure S5** (next page): The pollen diagram of Neumark-Nord 2, main curves; in full colour actual curves, in lighter colours: the same curves x 5.

**Figure S6**: Free vs Total D/L values of Asx, Glx, Ala and Val from bleached *Bithynia tentaculata* opercula from Neumark-Nord 2, compared with shells from UK sites correlated with MIS 5e, MIS 7 and MIS 9 (sample names indicate height in cm downward from the top of HP7).

**Figure S7**: Zijderveld diagrams of some representative samples in stratigraphic coordinates (the section underwent no tilting after deposition). Neu117: AF demagnetization (af) only; first Neu219 sample: AF demagnetization only; second Neu219 and Neu237 sample: thermal demagnetization (th) only. Neu253 and Neu255: AF demagnetization after thermal demagnetization up to 205°C. See text for more details.

**Figure S8**: Variable cut-off procedure after Vandamme (1994) for the HP7 and 210_296/297 samples. The scatter in ChRM directions caused by secular variation of the geomagnetic field are plotted in black (full symbols). Excursional directions are plotted in full red symbols (inclination positive or down) or open red symbols (inclination negative or up). Top panels HP7, lower panels excavation square section 210/296-297. VGPs (left-most panels) are centered on a vertical-up mean*.*
